# Topological data analysis of protein structure and inter/intra-molecular interaction changes attributable to amino acid mutations

**DOI:** 10.1016/j.csbj.2023.05.009

**Published:** 2023-05-09

**Authors:** Jun Koseki, Shuto Hayashi, Yasuhiro Kojima, Haruka Hirose, Teppei Shimamura

**Affiliations:** a Division of Systems Biology, Graduate School of Medicine, Nagoya University, Aichi 466-8550, Japan; b Department of Computational and Systems Biology, Medical Research Institute, Tokyo Medical and Dental University, Tokyo 113-8510, Japan; c Laboratory of Computational Life Science, National Cancer Center Research Institute, Tokyo 104-0045, Japan

**Keywords:** Topological data analysis, Persistent homology, Time course structural changes, Molecular dynamics, Amino acid mutations

## Abstract

The presence of some amino acid mutations in the amino acid sequence that determines a protein’s structure can significantly affect that 3D structure and its biological function. However, the effects upon structural and functional changes differ for each displaced amino acid, and it is very difficult to predict these changes in advance. Although computer simulations are very effective at predicting conformational changes, they struggle to determine whether the amino acid mutation of interest induces sufficient conformational changes, unless the researcher is a specialist in molecular structure calculations. Therefore, we created a framework that efficiently utilizes molecular dynamics and persistent homology methods to identify amino acid mutations that induce structural changes. We show that this framework can be used not only to predict conformational changes produced by amino acid mutations but also to extract groups of mutations that significantly alter similar molecular interactions, by capturing the resultant protein–protein interaction changes.

## Graphical Abstract

**Figure:**
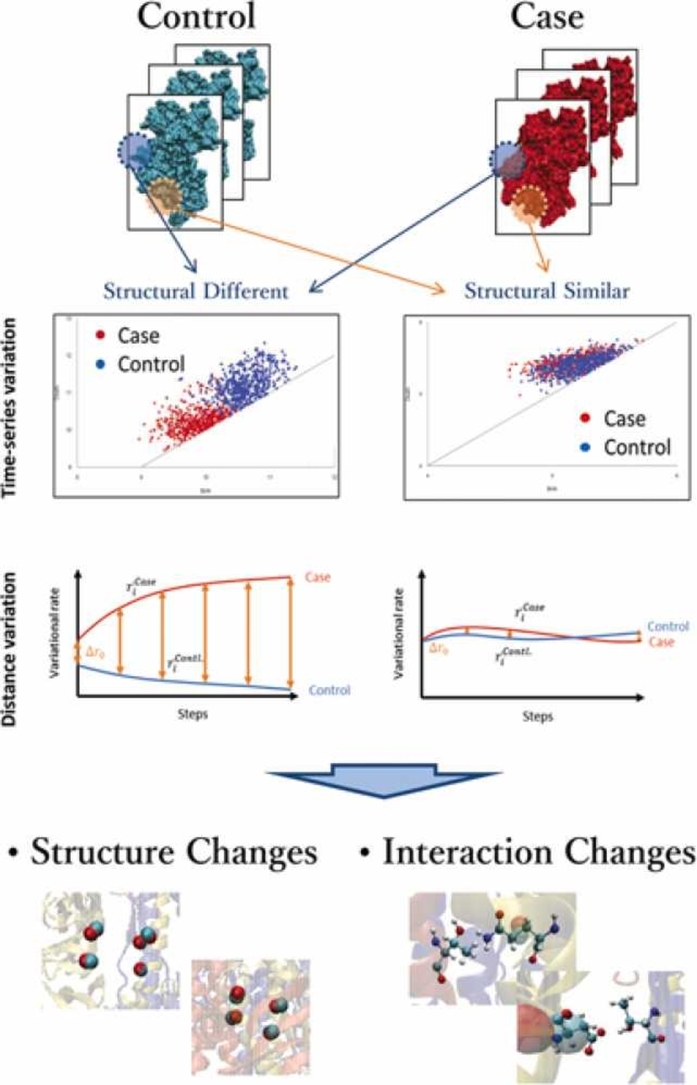

## 1 Introduction

The structural and dynamic properties of proteins are often related to their biological functions and molecular binding activities [1], [2]. In proteins, structural changes due to amino acid mutations can induce inter- and intra-molecular interaction changes, resulting in changes to protein conformation and potentially dramatic changes in biological function. Furthermore, amino acid mutations that accompany such protein conformational changes differ between different diseases (e.g., cancer [3], immunological diseases [4], and infectious diseases [5]), creating a diversity of conformational change patterns; however, many of the relationships between mutations and conformational changes remain unresolved. Elucidating the differences and diversity in structural and dynamic features of proteins is essential for understanding the biophysical mechanisms underlying them.

Traditional methods for identifying structural differences among proteins have included X-ray crystallography, nuclear magnetic resonance (NMR) analysis, and cryo-electron microscopy [1]. Whilst these methods can obtain snapshots of protein conformation at a few-angstrom-scale resolution, they cannot capture molecular behavior in vivo, which is affected by thermal oscillations attributable to body temperature and the surrounding environment. In addition, it is costly and impractical to observe this conformation for all amino acid mutations. The alanine-scanning method has also been used to identify amino acids involved in protein function. Although this method can examine the effect on function, it is difficult to observe how it affects the structure. On the other hand, theoretical methods such as molecular dynamics (MD) simulations provide important insights into the atomic motions that underlie the functions of many proteins; they also allow simulations to generate the structural ensembles that protein structures can adopt through mutations, as well as to compare the structural differences between proteins [6]. However, determining whether the protein conformational changes caused by thermal fluctuations during the simulation process are biologically essential differences, and extracting important structural features relevant to protein function, are non-trivial tasks and require expertize in structural and theoretical chemistry. Although several simple computer prediction methods have been developed to examine energy indices such as the degree to which the stability of the protein changes [7], [8], it is difficult to understand precisely how the structure changes.

*Dynamical Analysis of Interaction and Structural* c*hanges* (DAIS) is a computational framework that facilitates the automatic extraction of important structural features between two proteins without expertize or experience, by simultaneously extracting essential geometrical features and their dynamic changes from the patterns of protein conformational changes obtained in MD simulations. DAIS takes 4D atomic coordinates (consisting of a protein conformational time series obtained from MD simulations) as input and uses the persistent homology method [9] (a topological data analysis approach) to calculate 3D geometric and topological representations of protein structures (persistent diagrams) at each time point. The points plotted on the persistent diagram are the structural features of the protein. Each point is also a feature of a sub-structure within the protein. Therefore, these persistent diagrams for each time step are stitched together to facilitate sample-to-sample and time-to-time comparisons of protein structures from a geometric perspective. By tracking the behavior of holes (i.e., their mutation, disappearance, and creation) observed by the persistent homology method through MD simulations, DAIS can identify the sites of 3D structural changes and extract groups of atoms that form characteristic points with significantly differing structural properties. Furthermore, by using these groups of atoms (i.e., those that form feature points with different structural properties attributable to each amino acid mutation) as feature vectors, DAIS can visualize the diversity of protein conformational change patterns produced by multiple amino acid mutations. To demonstrate that DAIS can identify key structural and dynamic features, we performed a structural characterization of amino acid mutants observed in omicron mutant strains for the spike protein of severe acute respiratory syndrome coronavirus type 2 (SARS-CoV-2) [10], to extract the interaction changes pertinent to structural property changes. It is strongly expected that the computational framework developed in this study will help identify differences and diversity in the structural and dynamic features of proteins. Our code for the DAIS framework is freely available at https://github.com/jkoseki/DAIS.

## 2 Results

### 2.1 Conceptual view of DAIS for capturing structural changes

**Fig. 1:**
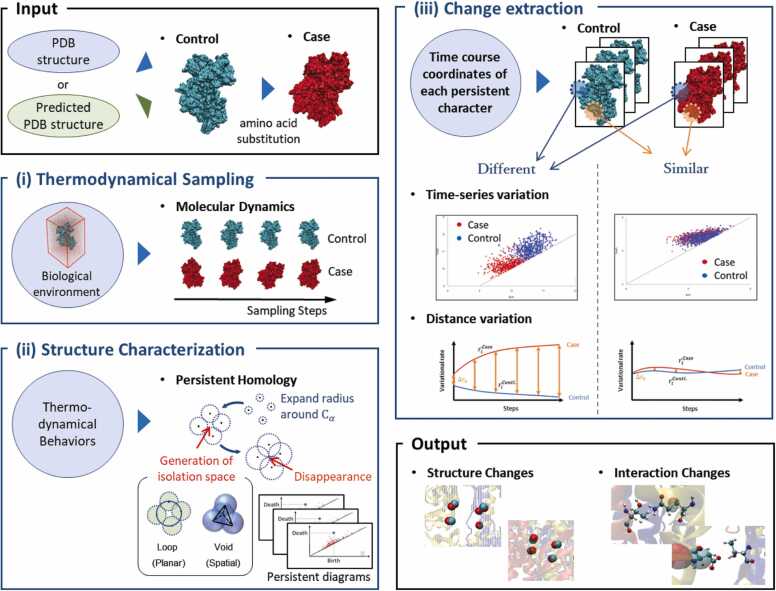
Schematic diagram of our analysis framework, divided into three stages: (i) thermodynamic sampling of proteins, (ii) structural characterization analyses of each structure using the persistent homology method, and (iii) extraction of characteristic conformational and interaction changes.

In this study, we constructed a framework called DAIS that effectively combines molecular dynamics and persistent homology methods to automatically identify the effects of partial molecular conformational changes (e.g., amino acid mutations) upon overall protein conformational changes and upon changes in intra- and intermolecular interactions. The specific process flow of this framework is as follows: To capture the structural changes induced by amino acid mutations and the internal interactions (e.g., hydrogen bonds) that propagate these structural changes, we combine MD and persistent homology methods. Our approach consists of three major stages (as shown in Fig. 1), and it takes as input the protein structure of reference (Control) and the amino acid mutation information for the target (Case) protein. The three stages are as follows: (i) thermodynamic sampling, (ii) structure characterization, and (iii) change extraction. DAIS outputs the sites where the structure and interactions have changed.

#### 2.1.1 Thermodynamic sampling

In the thermodynamic sampling process, the protein data bank (PDB) structure of the Control [e.g., a wild-type (WT) protein] is taken as an input to create a Case for a specific target (e.g., certain mutant protein structures), and MD is used to understand the molecular behavior of both in the in vivo environment. The prepared protein structures are stabilized in an aqueous solution via energy minimization, followed by protein conformational samplings extracted during the process of heating up from absolute zero temperature (0 K) to biological temperature (310 K). To better capture the differences between the two structures, we decided to create the Case structure by substituting amino acids into the Control structure. If the substituted mutation induces structural destabilization, a large conformational change is expected during the MD-induced temperature increase process. In other words, by simulating the process of temperature increase using molecular dynamics from a conformation in which Case and Control are nearly identical, the molecules move in a direction that stabilizes each other's structures, making it easier to observe structural differences. If the Control structure is unknown, it can be created using a prediction method such as Alphafold2. Even in this case, creating the case structure in the form of amino acid substitutions in the predicted control structure facilitates the extraction of sites at which the thermodynamic molecular behavior changes. For the reasons discussed above, we do not recommend that the structure of a mutant be generated using a prediction method such as Alphafold2, even if the missense mutant structure can be easily predicted using current methods.

#### 2.1.2 Structure characterization

The persistent homology method, a topological data analysis technique, is used to extract time-varying structural features from the thermodynamic sampling of protein structures; this method captures structural feature points according to the positional relationships between the data points of interest (in this case, the coordinate points of atoms). The presence of holes or cavities [isolation spaces shown in Fig. 1-(ii)] formed by the sphere centered at each data point of interest is a structural feature point. In particular, we track how the planar feature points (Loop) and spatial feature points (Void) observed using the persistent homology method behave on the persistent diagram (displacement, disappearance, and creation of corresponding feature points). The persistent homology method is independent of absolute coordinates and captures structural feature points according to the positional relationships (coordinates etc.) of the data points of interest; it is considered to be the best method for considering the features of systems in which the absolute coordinates of atoms change over time, as in this case [11]. Using this process, we obtain a time series of persistent diagrams, which plot the structural feature points for the Control and Case structures, respectively.

#### 2.1.3 Change extraction

By identifying behavioral differences between the corresponding structural feature points in Case and Control on the persistent diagram, we extract atoms that form feature points with significantly different structural characteristics and identify changes in the 3D structure. This adopts the concept that when we compare Control and Case structures and plot certain structural feature points in terms of their temporal movements on a persistent diagram, their distributions differ or overlap when structural differences do or do not occur, respectively. The difference in the distance between the percentage changes in the plots is used as an indicator of the magnitude of the molecular conformational change. In addition, we focus on hydrogen bonds in the vicinity of atoms that form the extracted feature points, and we calculate the rate of hydrogen bond formation between structural samplings; then, we extract interaction sites at which this bond formation rate differs significantly between Case and Control and extract the important hydrogen bonds involved in structural changes.

As long as we have tried with some proteins, in many cases (as shown in the following validation experiments), these processes alone were able to extract the structural and interaction changes; however, it is difficult to exclude structural regions that lack a specific secondary structure. In such cases, we believe that the use of the *Define Secondary Structure of Proteins* (DSSP) algorithm [12] is particularly important for extracting important skeletal structure changes, because DSSP allows us to analyze secondary structures and eliminate variation in regions lacking a specific structure.

### 2.2 Validation of structure change identification using DAIS

We show that, using our novel analytical framework DAIS, it is possible (i) to extract structural and hydrogen bond variations associated with amino acid changes and (ii) to analyze the diversity of protein–protein interactions at the interface, which are altered by amino acid mutations. In these validations, we use the structural changes produced by mutations in the spike protein of the SARS-CoV-2 virus and the protein–protein interaction between the receptor binding domain (RBD) of the spike protein and ACE2 as representative targets.

#### 2.2.1 Structural and intramolecular interaction changes

**Fig. 2:**
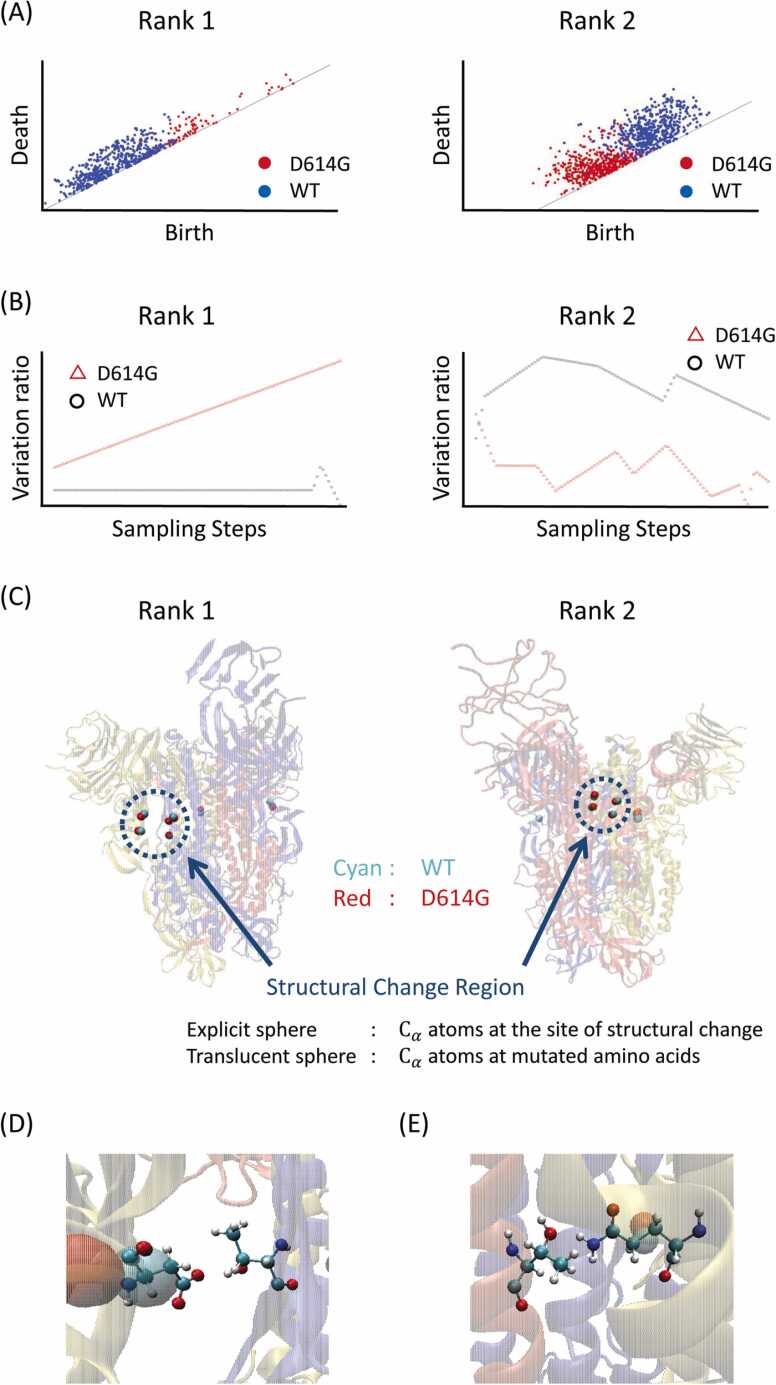
Comparison of WT and spiked proteins with the D614G mutation under our analysis framework: (A) The difference in persistent diagram distribution for the most-confirmed change (Rank 1) and second-most confirmed change (Rank 2) between D614G (red) and WT (blue). (B) Smoothed trajectory of the polar distance variation with respect to the accumulation step on the persistent diagram, with D614G and WT indicated by red triangles and black circles, respectively. (C) Structural change sites detected in Rank 1 and Rank 2. The explicit sphere in the dashed blue circle represents the C_α_ describing the structural change, and the other translucent spheres denote the C_α_ of the amino acid mutation sites. (D) Location of the most significant changes in the rate of hydrogen bond formation in the thermal vibration sampling of WT and D614G. (For interpretation of the references to color in this figure legend, the reader is referred to the web version of this article.)

For this validation, we use the D614G mutant [13], [14] and WT spike protein, whose conformational changes have already been confirmed via crystallographic analysis. We focus on the distribution of the feature tracking results for each point plotted on the persistent diagram between sampling steps. By applying our approach to this system, we can capture the distributions of the structural feature points on the persistent diagram that correspond to the D614G mutant and WT structures. The distributions observed here fall into two main categories, as shown in Fig. S1. The red and blue circles denote the structural characteristic points of Case and Control, respectively [i.e., when the distributions of D614G (Case) and WT (Control) are similar and when they differ]. Similar distributions mean that the structural features are similar and therefore no structural difference exists; meanwhile, if the distributions differ, the structural features are different and therefore the structure is considered to have changed. Here, we extracted important structural change points by scoring the distribution on the persistent diagram, as shown in Fig. S2; we discuss this further below. After excluding structural changes in regions without specific secondary structures using the DSSP package, we extracted large structural variations and identified the two locations shown in Fig. 2. (All structural changes extracted without the DSSP package are shown in Fig. S3.) Fig. 2A shows the different distributions of structural feature points between the D614G mutation and WT on the persistent diagram where structural changes were observed; Fig. 2B shows the smoothed change in the variation rate of the distance r in polar coordinate notation, where the distribution is transformed according to Fig. S2A. We confirm that these differences in the rate of variation are extracted as structural fluctuations that produce large differences, as shown in Fig. S2B. The sites of structural change corresponding to these distribution changes are shown in Fig. 2C. The explicitly indicated spheres surrounded by dashed blue lines refer to carbon atoms at the alpha position ($C_{\alpha }$: alpha-carbons; CAs) of amino acids, which form the feature points where structural changes are expected; the translucent spheres refer to the CAs of displaced amino acids. Our approach allows us to automatically extract regions of conformational change in the hydrogen bonding site that are hidden by the mutation (Rank 1) and regions of thermal vibration variation at the trimer–binding interface of the spiked protein (Rank 2). Fig. 2D and E can also be extracted as hydrogen bonding changes that influence these structural changes, without the need for user judgment. The hydrogen bond shown in Fig. 2D is the hydrogen bond formed by 614D, and Fig. 2E shows the hydrogen bond at the trimer interface. On the other hand, when our approach was applied to mutants without the D614G mutation, the structural changes shown in Fig. S4A and B were extracted. Fig. S4C was excluded using the DSSP package. The structural changes around the amino acid mutation sites were extracted; however, the hydrogen bonds that contribute to the structural variation or that propagate throughout the structure were excluded. By applying our method to the D614G mutant in which the conformational change occurred, we show that the computer—which does not know the correct answer—is able to extract the hydrogen bonding site that (i) is cleaved by the mutation, (ii) is therefore more prone to fluctuations, and (iii) in turn increases the likelihood of fluctuations in the interfacial portion of the trimer. In other words, this framework strongly reproduces the experimental observations. On the other hand, for variants without large conformational changes, small conformational changes around the mutant amino acids emerge, though these did not alter the internal hydrogen bonds, and we predicted that the conformational changes would not propagate. For the D614G mutation, DUET [7], one of the computer prediction methods for amino acid mutations, predicts that the stability change (ΔΔG) due to the mutation is destabilizing at − 0.892 kcal/mol, showing the same trend as DAIS.

#### 2.2.2 Protein–protein interaction changes

**Fig. 3:**
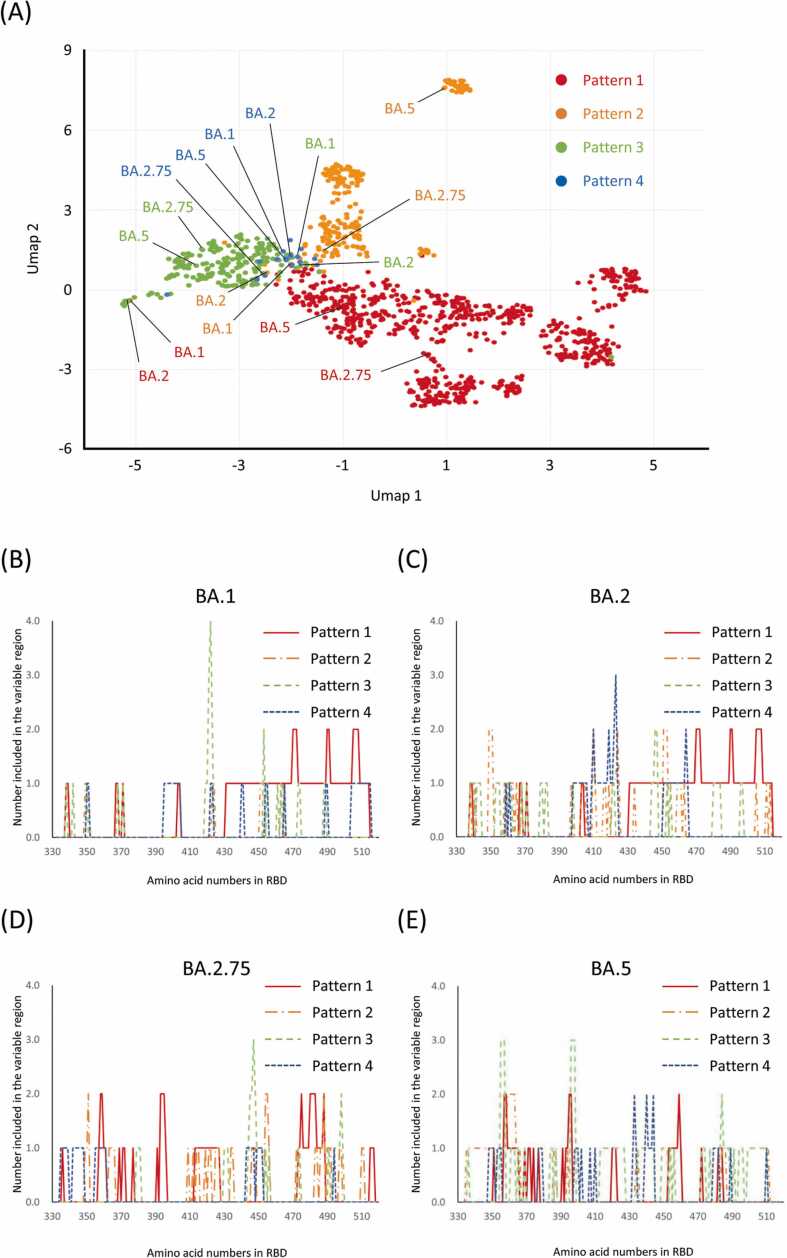
Conformational variation pattern analysis in RBD and ACE2 binding of spike protein: (A) UMAP projection of the structural variation induced by the mutation pattern in each binding pattern. Patterns 1, 2, 3, and 4 are indicated in red, orange, yellow-green, and blue circles, respectively. (B–E) Counts of conformationally variable amino acids in the RBD in BA.1, 2, 2.75, and 5 variants. (For interpretation of the references to color in this figure legend, the reader is referred to the web version of this article.)

Then, we applied our framework to changes in protein–protein interactions and binding conformational changes, to analyze binding differences between RBD and ACE2 in the spike protein. In particular, we generated binding conformations between RBD and ACE2 for various mutants (e.g., BA.1, BA.2, BA.2.75, and BA.5) and focused on their relationships. In the binding between the RBD of the spike protein and ACE2, we found two states: "up" (in which the RBD portion rises) and "down" (in which the RBD portion falls) [15]. We classified our binding conformation patterns into Patterns 1–4 depending on the binding orientation to ACE2. On the other hand, Greaney et al. classified our Binding Patterns 3 and 4 into the same class (Class 3) when using antibody escape as the classification criteria [16]. Here (see Fig. 3A), the differences in the binding patterns of RBD and ACE2 (Patterns 1–4 in Fig. S5) indicate that each mutation confers a different binding stable structure between proteins on the UMAP area. The proteins are clustered according to their binding patterns. The number of conformational change counts for each amino acid, as observed under the persistent homology method, is shown in Supplemental Table 1. Fig. 3A shows that we can see that the effect of the dynamic coupling variation produced by Pattern 4 can be captured as part of the Pattern 3 distribution. Our computational results support the results of that study regarding antibody escapes. Here, we recaptured the RBD and ACE2 in terms of their binding orientations. In Fig. S6A, amino acid numbers 332–380 are shown in green, 381–429 in purple, 430–469 in yellow, and 370–518 in pink. As shown Fig. S6C, Patterns 3 and 4 are bound to RBDs in the DOWN state; however, the binding orientations differ, and they intersect many of the green (332−380) and yellow (430−469) areas. MD calculations also show that the molecular vibrations in Pattern 3 are oriented in a bond orientation direction similar to that in Pattern 4, and it seems very reasonable that the effects upon bond conformational changes are similar.

**Table 1:** Amino acid mutations observed in RBD in BA.1, BA.2, BA.2.75, and BA.5, as well as their respective prevalences.

BA.1				BA.2				BA.2.75				BA.5			
Mut.			Prevalence	Mut.			Prevalence	Mut.			Prevalence	Mut.			Prevalence
G	339	D	88.5	G	339	D	96.3	G	339	H	95.5	G	339	D	99.3
R	346	K	0.5												
S	371	L	81.8	S	371	F	95.9	S	371	F	96.7	S	371	F	99.2
S	373	P	82.3	S	373	P	96.4	S	373	P	95.8	S	373	P	100.0
S	375	F	82.4	S	375	F	96.1	S	375	F	93.9	S	375	F	100.0
				T	376	A	95.7	T	376	A	94.2	T	376	A	100.0
				D	405	N	96.9	D	405	N	98.6	D	405	N	99.9
				R	408	S	95.8	R	408	S	93.7	R	408	S	94.4
K	417	N	54.4	K	417	N	95.4	K	417	N	89.4	K	417	N	91.3
N	440	K	56.7	N	440	K	87.3	N	440	K	91.5	N	440	K	90.0
G	446	S	57.6					G	446	S	93.0				
L	452	R	0.5									L	452	R	97.8
								N	460	K	93.2				
S	477	N	88.0	S	477	N	94.3	S	477	N	89.0	S	477	N	99.4
T	478	K	88.3	T	478	K	94.4	T	478	K	88.7	T	478	K	99.4
E	484	A	87.9	E	484	A	94.5	E	484	A	88.9	E	484	A	99.5
												F	486	V	99.7
Q	493	R	88.5	Q	493	R	94.5								
G	496	S	87.9												
Q	498	R	87.8	Q	498	R	93.6	Q	498	R	85.9	Q	498	R	98.4
N	501	Y	88.1	N	501	Y	93.8	N	501	Y	86.8	N	501	Y	98.8
Y	505	H	87.8	Y	505	H	93.5	Y	505	H	85.7	Y	505	H	98.4

**Fig. 4:**
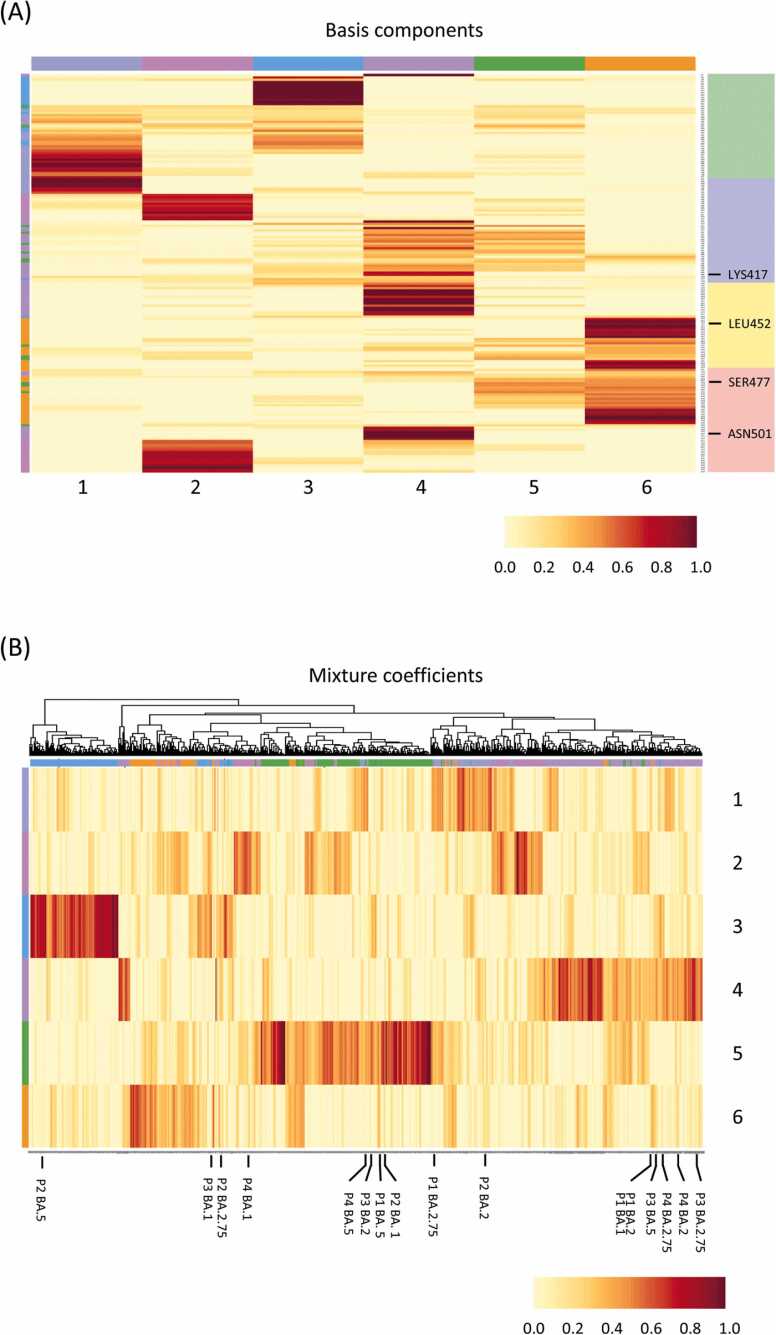
Feature analysis using non-negative matrix factorization of conformational variation in RBD and ACE2 binding of spike proteins. (A) and (B) denote basis components and mixture coefficients, respectively. The color map behind the amino acid names on the right axis in (A) shows the RBD structure codes by amino acid number; Green, Iceblue, Yellow, and Pink refer to amino acid numbers 332–380, 381–429, 430–469, and 470–518, respectively. (For interpretation of the references to color in this figure legend, the reader is referred to the web version of this article.)

On the other hand, the BA.1 and BA.2 binding structures of Pattern 1 are plotted at a significant distance from the distribution of structural variation given by the other Pattern 1 mutant binding structure groups. Fig. 3B–E show the counts for amino acids determined to be conformationally altered during MD sampling of the RBD and ACE2 complex for BA.1, BA.2, BA.2.75, and BA.5, respectively, as compared to the WT complex. In the complex structure of Pattern 1 for both BA.1 and BA.2, the counts increase around amino acid numbers 470, 490, and 505, and they are also widely counted before and after those numbers. In fact, in the SARS-CoV-2 virus mutation, structural mutations are identified in the regions where amino acid arrangements such as S477N and N501Y (which are heavy research focuses) are present. Table 1 shows the mutation patterns within the RBD and the mutation prevalence in each lineage. In BA.1 and BA.2, the crystal structure of the bond in Pattern 1 is confirmed. The difference between BA.1 and BA.2 is that the RBD amino acid number (from 330 to 370) is prone to large variations in the binding structure to ACE2, even though the number of mutations is identical. This trend continues in BA.2.75. and BA.5; however, the fluctuations around 490 and 505 show a decrease. As seen above, Pattern 1 for these mutants tends to indicate structural changes in the region beyond amino acid number 430. The amino acid number 430 and beyond are denoted in pink in Fig. S6. On the other hand, the recently observed BA.2.75 and BA.5, in contrast to BA.1 and BA.2, exhibit reduced structural effects upon amino acid numbers 430 and beyond but increased effects on structural changes from 330 to ∼400. This may account for the differences in binding stability between BA.2.75 and BA.5 compared to BA.1 and BA.2. The relationship between each mutant and the amino acids that induce further structural changes, as characterized using non-negative matrix factorization (NMF) [16], is shown in Fig. 4 and S7. For example, the Pattern Structures BA.1 and BA.2 contains Components 2, 4, and 6 of the basis components. On the other hand, BA.2.75 and BA.5 in Pattern 1 do not markedly contain Basis Components 2, 4, or 6 but strongly exhibit Component 5. These results have the same meaning as in Fig. 3A and B. In Fig. 4B, we can see and capture the BA variants with similar compositions. For example, BA.1 and BA.2 in Pattern 1 induce structural variations very similar to those of BA.5 in Pattern 3 and BA.2.75 in Pattern 4. Fig. S6 shows the amino acid numbers and positions of the RBD sites, which are roughly classified and color-coded according to the count peaks in Fig. 4B–E. This reveals amino-acid-mutation-induced changes in the interactions between the two proteins, and they show that it is possible to extract the amino acid sites that produce significant changes in the interaction. Thus, the combination of MD and persistent homology methods constitutes a very effective approach for qualitatively predicting the structural effects of amino acid mutations as well as their effects on protein–protein interactions.

## 3 Discussion

Understanding the changes in the tertiary structures of proteins and their functions (generated as a result of genetic-mutation-induced changes in the amino acid sequence) is an extremely important field in the life sciences. In recent years, topology-based structural analysis methods have been focused upon and applied in many fields. However, it remains difficult to capture dynamical structural and functional changes in real life. In this paper, we proposed DAIS, a novel framework that can analyze the diversity of multiple variants with various amino acid mutations, and we demonstrated its utility. DAIS is a very powerful framework when the possibility of multiple variants needs to be considered, and it is extensible for the user's purposes. We were able to show that our framework not only makes it possible to extract the areas in which the protein structure differs when comparing the Control and Case but also the hydrogen bonds that are responsible for the conformational change or that are important for the propagation thereof. In addition, we can characterize the effects of amino acid variants upon protein–protein interactions and identify which variants are similar. In this framework, protein conformational change extraction focuses on CA (the skeletal structural carbon), and the scoring of conformational changes between comparisons prevents agreement between the molecular vibration ranges in MD. This means that the initial velocity dependence of MD can be eliminated; hence, we need not apply MD to the same protein multiple times and can thereby reduce the computational cost. Furthermore, under this framework, when conducting persistent homology assessments, the positional information of the CAs that form each structural feature point is retained, making it possible to link them to the hydrogen bond change analysis results, as was done here. Although we are currently unable to extract changes in molecular interactions (other than hydrogen bonding), this is a very important point for future extension to the analysis of physical interactions, including van der Waals interactions within or between molecules. While conventional computer prediction methods can show whether an amino acid mutation stabilizes or destabilizes a protein with an energy index, our framework has the advantage of being able to present the actual conformational change caused by the mutation, and also extract conformational changes propagating directly from the mutation site. Using this framework, we can predict changes in protein–protein or protein–binding molecule interactions as well as their similarities among various mutants. Thus, in the case of viral infections, it will be possible to predict which mutations will produce a change in virulence; furthermore, in the case of drug target proteins, it will be possible to determine which mutations may produce resistance to the drug.

## 4 Methods

### 4.1 Preparation of protein initial structures and thermodynamic structure sampling

#### 4.1.1 Examining the impact of the D614G mutation

To capture the effects of the D614G mutation [present in 94 % of the omicron variants (B.1.1.529)], we created WT and mutant structures [according to the crystal structures (PDB ID: 6XS6 [13])]. The crystal structure (PDB ID: 6XR8 [17]) was used to compare WT against mutants (Q52R, E484K, Q677H, F888L) that do not contain D614G and exhibit no significant structural changes. Each structure was prepared using the PrepWizard program [18] provided by the Schrödinger Corporation, to identify the disulfide bonding sites from the protein backbone structure and to optimize the hydrogen bonding network (including hydrogen addition and the protonation state of histidine). Some counter ions (sodium ion: Na^+^) were inserted to neutralize the overall charge of the system, and 12-Å MD cells filled with water molecules were created around each protein. Energy minimization calculations were performed upon these structures to stabilize the overall system structure. Using the relaxed structures as the initial structures, MD simulations were performed to sample the fluctuations in protein structure. In this calculation, 500,000 steps were performed with a time step of 0.2 fs, and the coordination of the system was accumulated every 1000 steps. These molecular mechanical calculations were performed using the AMBER 18 program package [19]. The AMBER 99SB [20] and TIP3P [21] force fields were employed for spike proteins and water molecules, respectively.

#### 4.1.2 Verification of the effects of mutations at the RBD site

The spike protein structures of some mutants were created by amino acid substitutions based on the crystal structure (PDB ID: 6ZGI [22]). The mutation-information was obtained from Outbreak.info, a SARS-CoV-2 database provided by Scripps Research. Similar to the D614G mutant protein, these proteins were also subjected to MD, to fully stabilize the structure to a thermodynamic equilibrium. By superimposing the RBD portion of the crystal structure that captured the ACE2 and RBD binding (PDB ID: 6XCM [23], 6XCN [23], 7K90 [24], 7K8S [24], 7K8T [24], 7K8X [24], 7K8Y [24], 7K8Z [24], and 7K8V [24], as shown in Supplemental Table 2) and the RBD portion of the thermally equilibrated spike protein structure, we created several patterns for certain variants (e.g., BA.1, BA.2, BA.2.75, and BA.5) and the initial structure of the ACE2 binding site, as shown in Fig. S5 (3 classes; 4 patterns; 12 conformations). The crystal structures referred to here are the binding structures of different antibody classes, as classified in existing studies. In this calculation, we extracted amino acid mutations with high antibody evasion rates and combined them to create a total of 1171 mutant variants (Pattern 1: 708, Pattern 2: 229, Pattern 3: 218, Pattern 4: 16). These structures were used to consider the binding structures with up or down RBDs. Again, as with the previous D614G mutation, we sampled 500 thermodynamic conformations for the binding structure, using MD calculations.

### 4.2 Analysis of structural and interaction changes using DAIS

A flowchart outlining this framework is shown in Fig. S8. The details of each step are described below.

#### 4.2.1 Topological data analysis for thermodynamical conformations

To analyze the structural and intramolecular interaction changes produced by amino acid mutations in proteins, we extracted carbons at the alpha position ($C_{\alpha }$: alpha-carbons, CAs) of the protein backbone from the thermodynamic configurations (sampled using MD calculations). We randomly selected 500 representative CAs from amongst them and applied the persistent homology method [a topological data analysis (TDA) method] using these CAs. The persistent homology method captures structural features from the loops (dimension 1) and voids (dimension 2) created when the radius of a sphere centered on the n structural components (connected components: dimension 0) of interest was gradually increased. Persistent diagrams are often used to visualize these structural features. In these diagrams, each structural feature point is plotted on the horizontal axis as the radius when the feature point is created (Birth) and upon the vertical axis as the radius when the feature point disappears (Death). TDA analysis was performed on the structure of each sampled target protein, and the structural feature points on the persistent diagram were identified between different time points or between proteins, to be compared using the following homology formula [25]:

$$\mathrm{Sim}\left({c_{\alpha }}_{i}^{p},{c_{\alpha }}_{j}^{q}\right)=\left\{\begin{array}{cc} \frac{\left|{c_{\alpha }}_{i}^{p}\cap {c_{\alpha }}_{j}^{q}\right|}{\max\left(\left|{c_{\alpha }}_{i}^{p}\right|,\left|{c_{\alpha }}_{j}^{q}\right|\right)} & \;\mathrm{if}\;\frac{\left|{c_{\alpha }}_{i}^{p}\cap {c_{\alpha }}_{j}^{q}\right|}{\max\left(\left|{c_{\alpha }}_{i}^{p}\right|,\left|{c_{\alpha }}_{j}^{q}\right|\right)}>\delta \\ 0 & \mathrm{otherwise} \end{array}\right)$$

Here, for the comparative structural features p and q in different time series i and j (i≠j, if p=q), the alpha-carbon groups comprising each structural feature are denoted as ${c_{\alpha }}_{i}^{p}$ and ${c_{\alpha }}_{j}^{q}$, respectively. δ is the threshold value for considering homology; in this study, we employed δ=0.75. In other words, if the threshold value δ is greater than 0.75, ${c_{\alpha }}_{i}^{p}$ and ${c_{\alpha }}_{j}^{q}$ are taken to be structural features corresponding to the same structural site. For all structural features identified as identical, the coordinate variation over time on the persistent diagram was plotted. In this study, all TDA analyses for 500 conformations of each target protein were performed with R package 3.6.3 and the TDA package [26].

#### 4.2.2 Analysis of important structural changes

To capture the time variation of each structural feature plotted on the persistent diagram, the abscissa of this diagram was kept as the Birth radius, and the value of the ordinate was set as the difference between the Death and Birth radii. This was then transformed into a polar coordinate system, to extract the time variation of the distance component, as shown in Fig S2. Then, we divided this by the initial distance to obtain the rate of change in the distance component ($r_{i}^{\mathrm{Contl}.},r_{j}^{\mathrm{Case}}$), so that we could uniformly compare the amount of change between structural feature points. Then, the change in the distance variation ratio over time was smoothed (${\overset{®}{r}}_{i}^{\mathrm{Contl}.},{\overset{®}{r}}_{i}^{\mathrm{Case}}$). For each structural feature of the proteins to be compared (Contl. and; e.g., WT and mutant, respectively), the difference in the rate of structural change over time $\left(∆r_{i}={\overset{®}{r}}_{i}^{\mathrm{Case}}-{\overset{®}{r}}_{i}^{\mathrm{Contl}.}\right)$ was calculated. As a score to determine the importance of the structural changes (ID score), we defined the absolute value of the sum of the differences between the previous structural change percentages ($\left|\sum_{i}∆r_{i}\right|$). Here, to increase the sensitivity of the structural changes to be extracted, structural features belonging to the 1st percentile or below and 99th percentile or above were excluded from the distribution of ID scores. In addition, as shown in Fig S2, structural features belonging to the 5th–95th percentile in the ID score distribution were excluded as having small differences in their variability between comparisons.

#### 4.2.3 Analysis of important intramolecular interaction changes

To investigate changes in hydrogen bonding, one of the interactions between side chains that induces or propagates changes in skeletal structure, we first extracted the CA-containing amino acid atoms within 10 Å of each of the CA groups comprising the structural feature of interest. We searched for oxygen or nitrogen atoms that were at least 1.5–2.2 Å away from the hydrogen bonded to the nitrogen or oxygen (hydrogen bonding distance: 1.5≤R≤2.2), and we extracted hydrogen bonds when the angle between these three atoms (θ) exceeded 160° (160°≤θ). We adopted this threshold because hydrogen bonds formed by oxygen and nitrogen are classified as strong, and it has been reported that the distance between hydrogen bonds is 1.5≤R≤2.2 Å and that the angle is 160°≤θ [27]. Here, the percentages of hydrogen bonding formation times were calculated using the MD sampling structures; those with a large difference between targets were extracted.

### 4.3 Statistical analysis of binding structural change patterns between ACE2 and RBD of spike protein

The binding structures of ACE2, the WT RBDs, and each mutant spike protein sampled using MD simulations were compared, and the number of CAs at sites where structural changes were identified (under the persistent homology method) were counted. The dynamic changes given by all the generated mutant complex structures were visualized using the UMAP package [28] in R. We further used the NMF package [16] to characterize which structural changes each mutant was likely to induce, and to analyze what relationships were present between mutations. The rank decision used in the NMF was based on the decreasing "cophenetic" level.

## Author contributions

**JK, SH,** and **TS** designed this research and undertook program creation and variation analyses. **JK, SH, YK, HH**, and **TS** discussed improvements to the research. **JK, SH,** and **TS** identified and gathered information on SARS-CoV-2 virus mutations. **JK, SH**, and **TS** wrote the manuscript. All authors have read and approved the manuscript.

## Declaration of Competing Interest

The authors declare no conflict of interest.
