# Supplementary material for: Topological data analysis of protein structure and inter/intra-molecular interaction changes attributable to amino acid mutations
Source: Comput Struct Biotechnol J. 2023 May 9;21:2950–9. doi: 10.1016/j.csbj.2023.05.009 (PMC10205437; doi:10.1016/j.csbj.2023.05.009)
Supplement: Supplementary file 1 — Supplementary material [file mmc1.docx]

**Topological data analysis of protein structure and inter/intra- molecular interaction attributable to amino acid mutations**

Jun Koseki^1,†*^, Shuto Hayashi^1,†^, Yasuhiro Kojima^1^, Haruka Hirose^1^, Hyunha Nam^1^, Teppei Shimamura^1,*^

1 Division of Systems Biology, Graduate School of Medicine, Nagoya University, Aichi, 466-8550, Japan.

*Correspondence

^†^Equally Contributed

Division of Systems Biology

Graduate School of Medicine, Nagoya University

65, Tsurumai cho, Showa ku, Nagoyashi, Aichi 466-8550, Japan.

Tel: +81-52-744-1980; Fax: +81-52-744-2029

E-mail: [jkoseki@med.nagoya-u.ac.jp](mailto:jkoseki@med.nagoya-u.ac.jp)

E-mail: [shimamura@med.nagoya-u.ac.jp](mailto:shimamura@med.nagoya-u.ac.jp)

Keywords: Topological data analysis, Persistent homology, Time course structural changes, Molecular dynamics, Amino acid mutations

**Supplemental Figures**

**
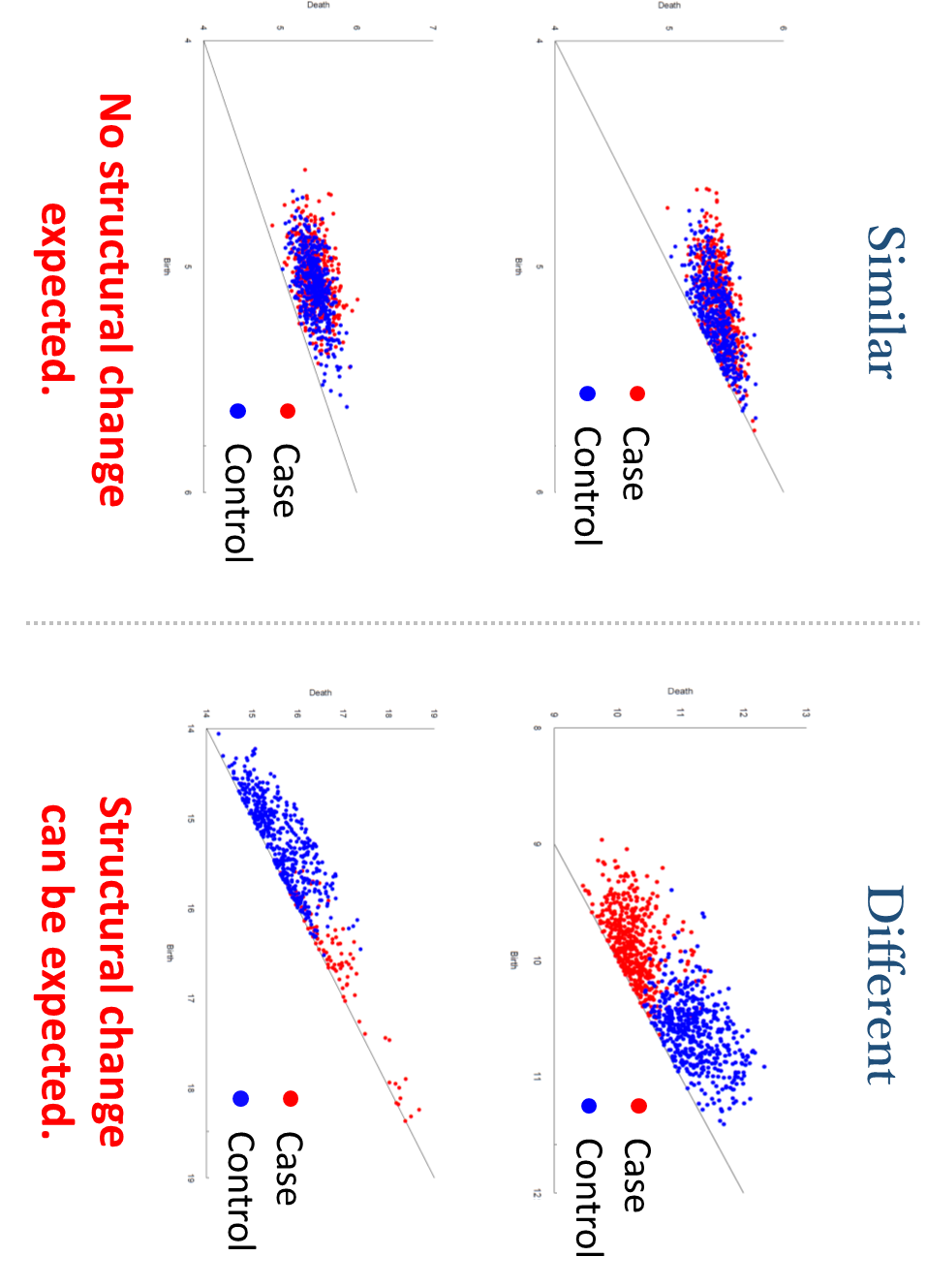
**

**Figure S1:** The difference in the distribution of the persistent diagram. Distribution overlap between Case and Control. when no difference in structure occurs (left) and when a difference in structure occurs (right).


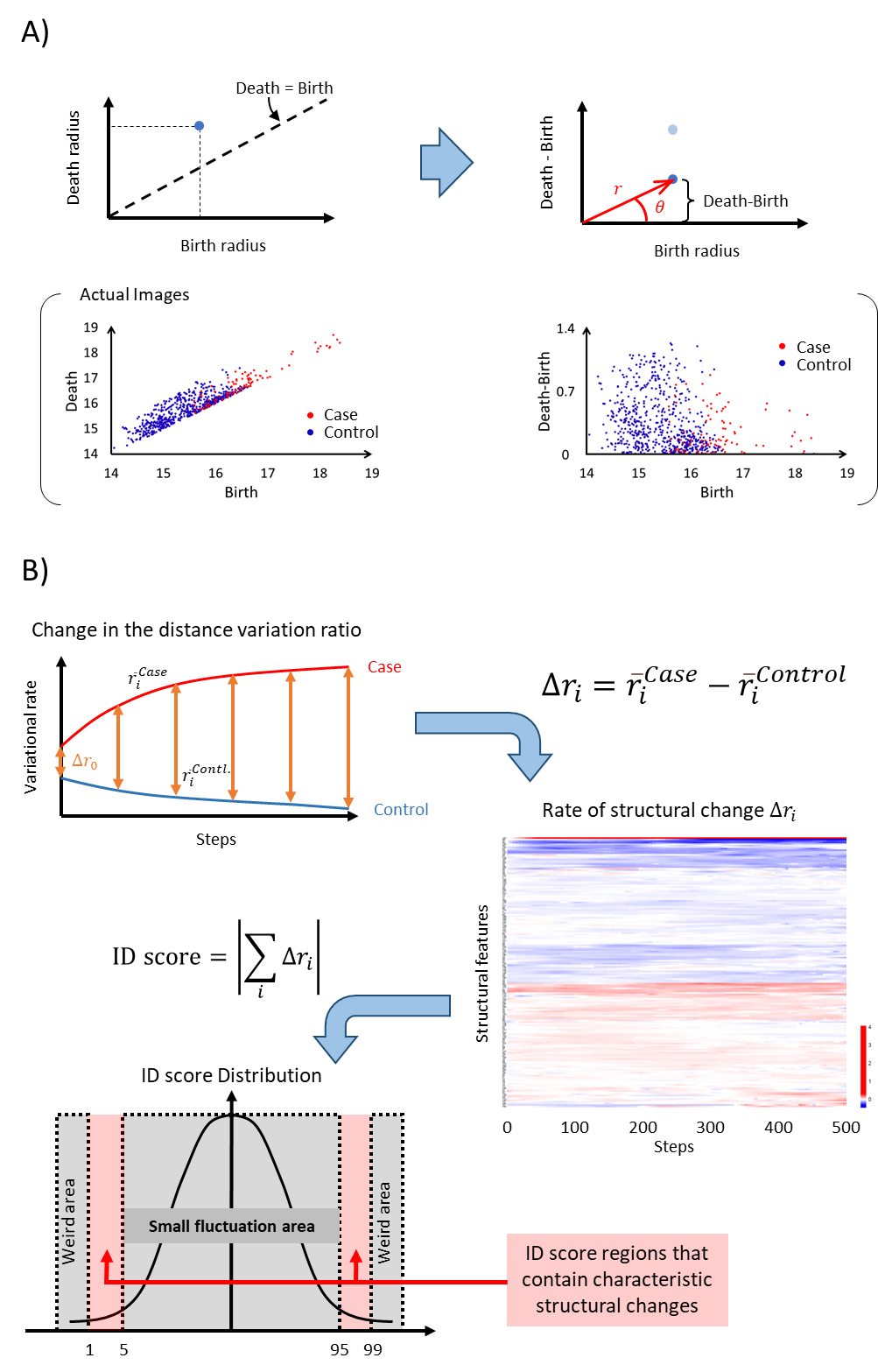


**Figure S2:** Schematic diagram for observing the variation of feature points on a persistent diagram. (A) The concepts of translation and polarization of persistent diagrams. (B) Time-varying differences in polar distances and scoring of the variation.


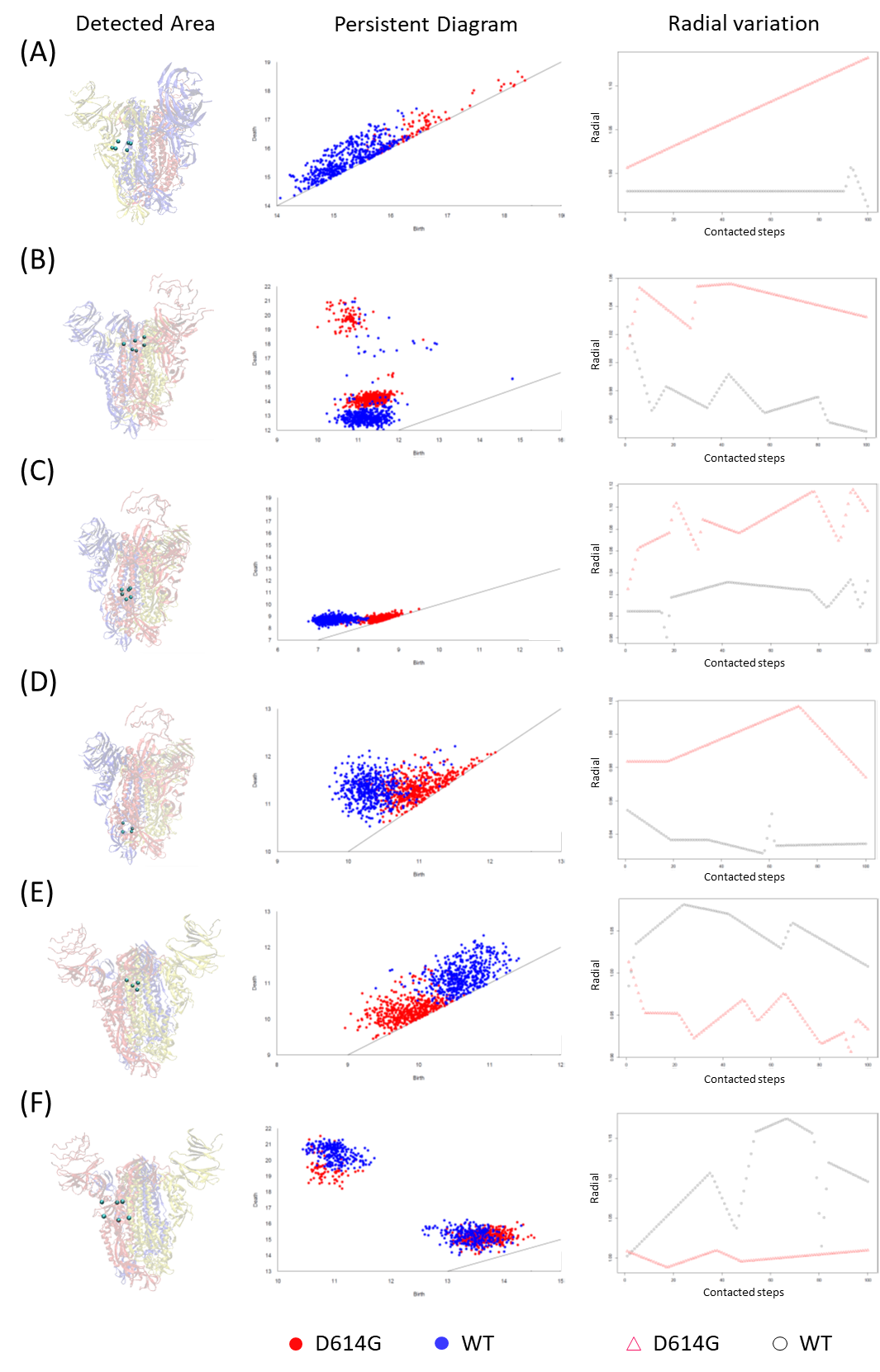


**Figure S3:** All structural changes detected by score in compared with WT and D614G. These structural changes include variations in flexible areas that do not have a specific secondary structure.


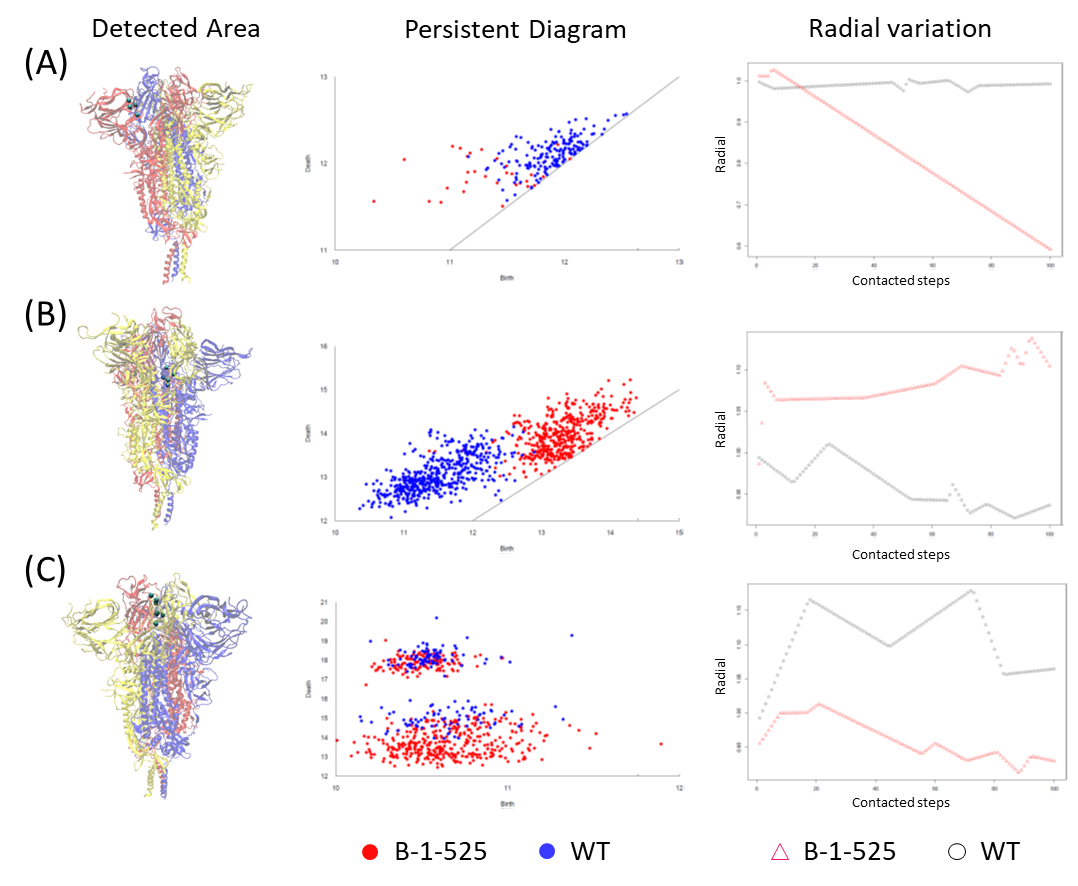


**Figure S4:** All structural changes detected by score in compared with WT and the mutant variant. These structural changes include variations in flexible areas that do not have a specific secondary structure.


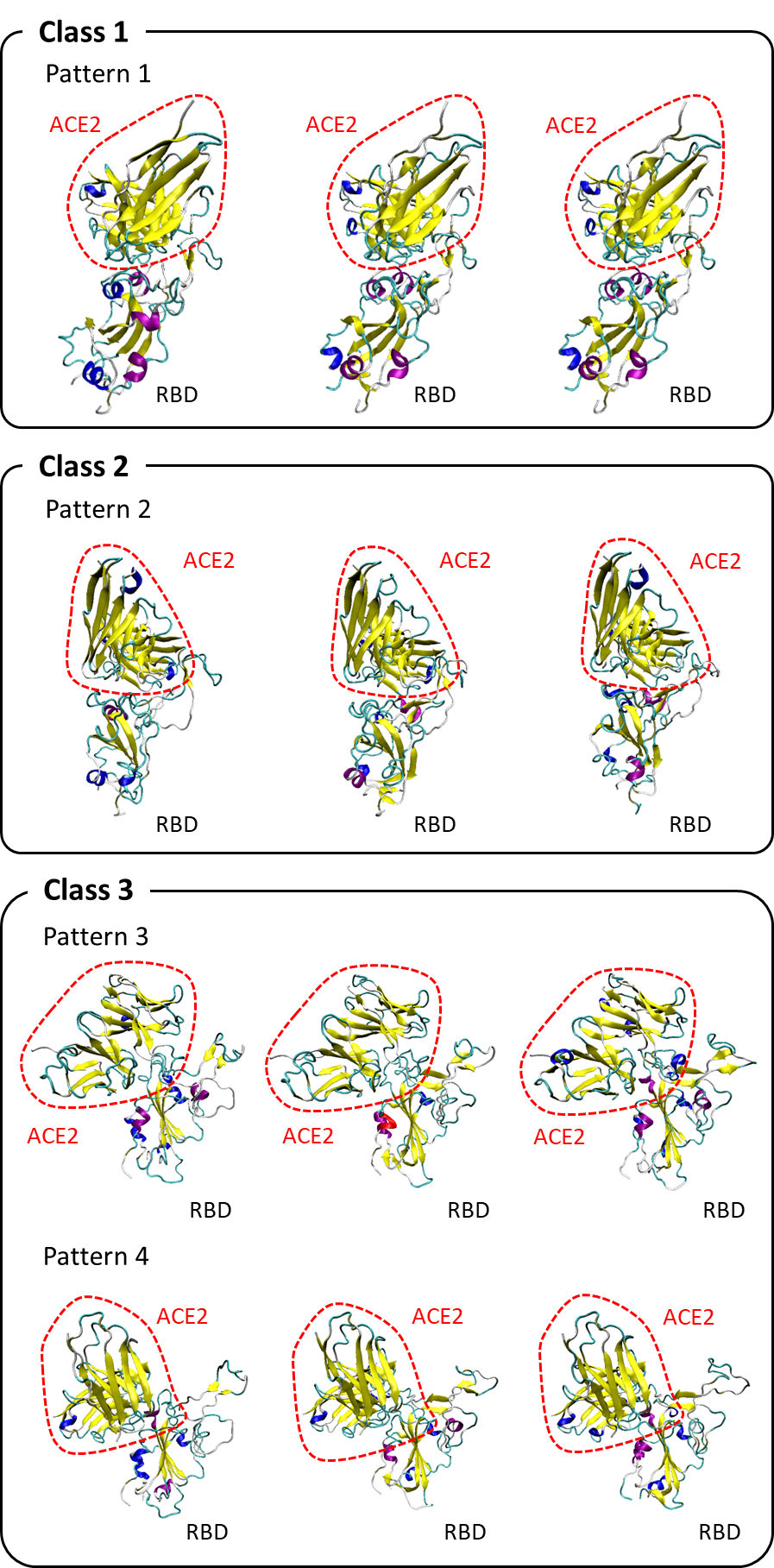


**Figure S5:** Classification in existing studies based on antibody escapes and our binding patterning based on binding orientation.


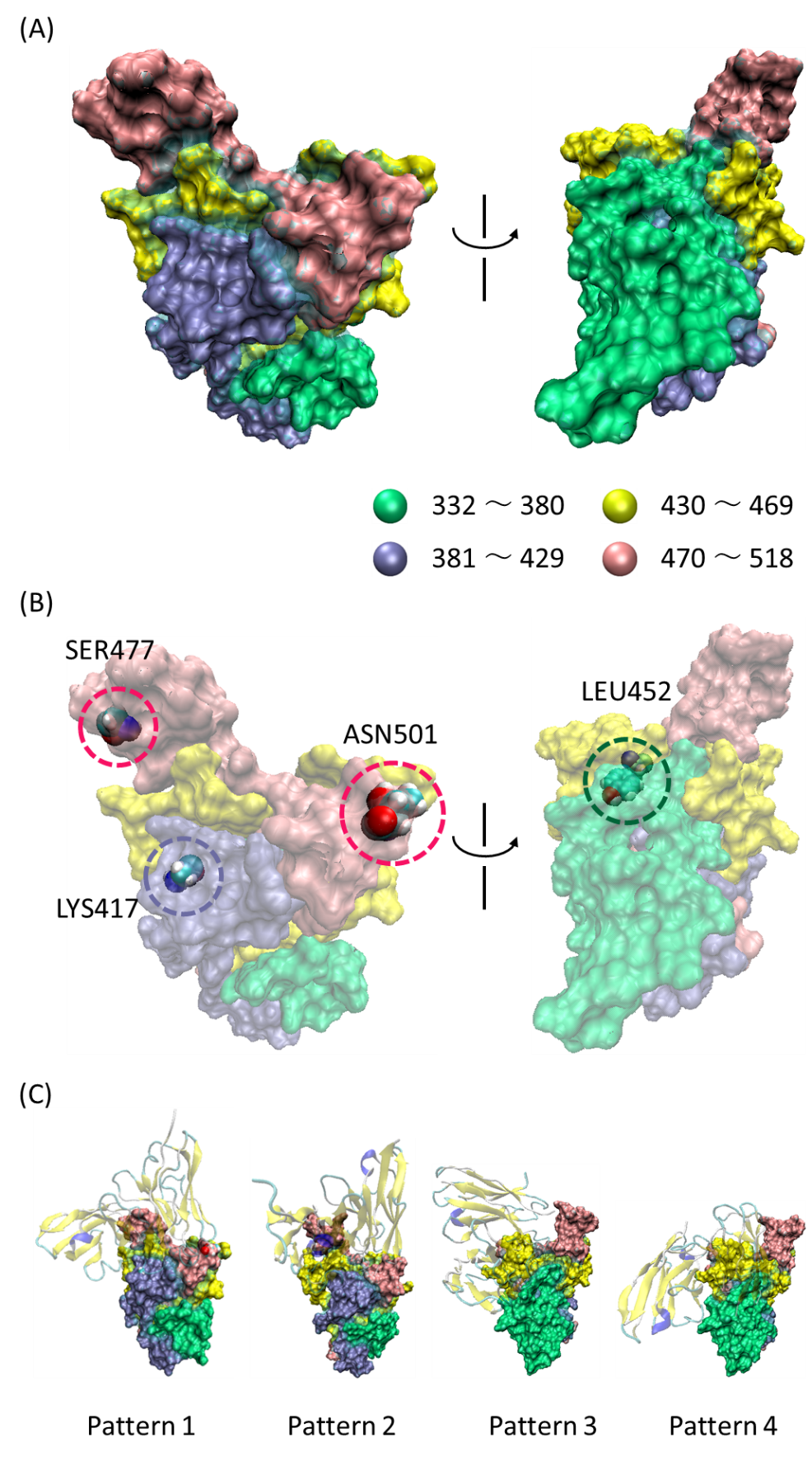


**Figure S6:** These color map shows the structural coding of RBD by amino acid number. (A) The RBD structure codes by amino acid number; Green, Iceblue, Yellow, and Pink refer to amino acid numbers 332-380, 381-429, 430-469, and 470-518, respectively. (B) The amino acid positions where mutations of particular interest occur are indicated by van der Waals spheres. (C) The four patterns of ACE2-RBD binding in relation to the interaction interface.


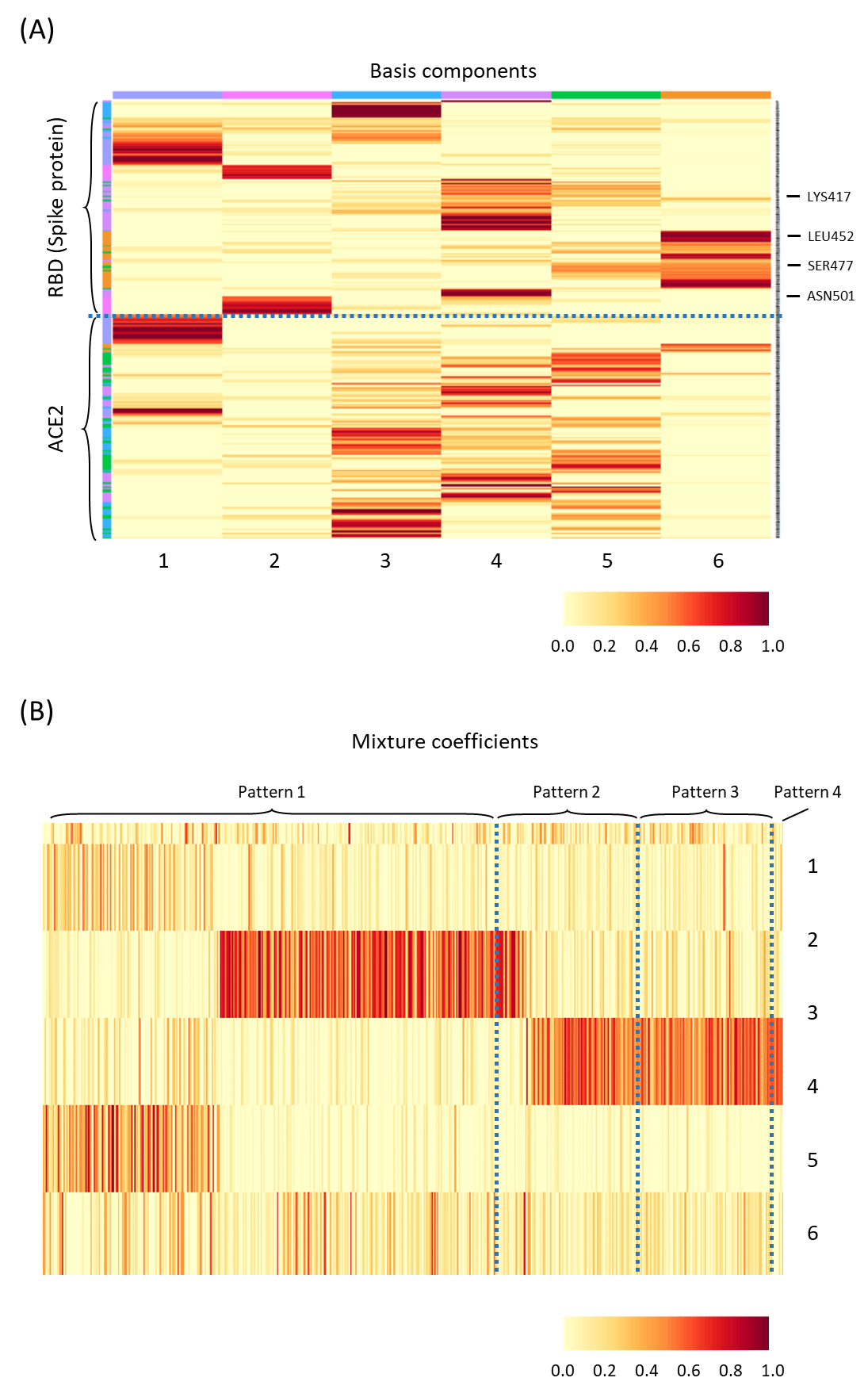


**Figure S7:** Feature analysis using nonnegative matrix factorization of conformational variation in RBD and ACE2 binding of spike proteins. (A) Basis component when including structural variation in ACE2. (B) Mixture Coefficients shown for each pattern.


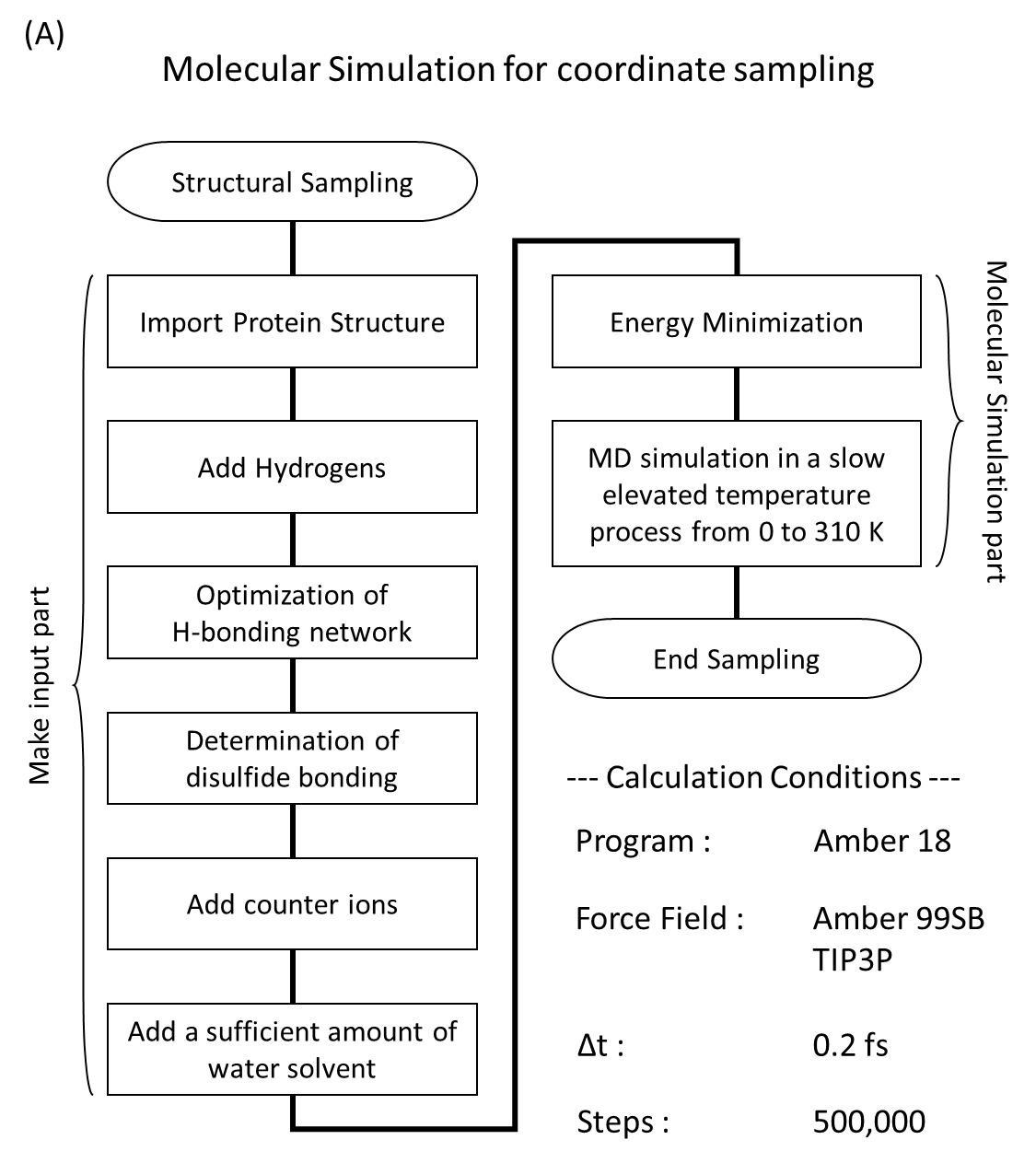


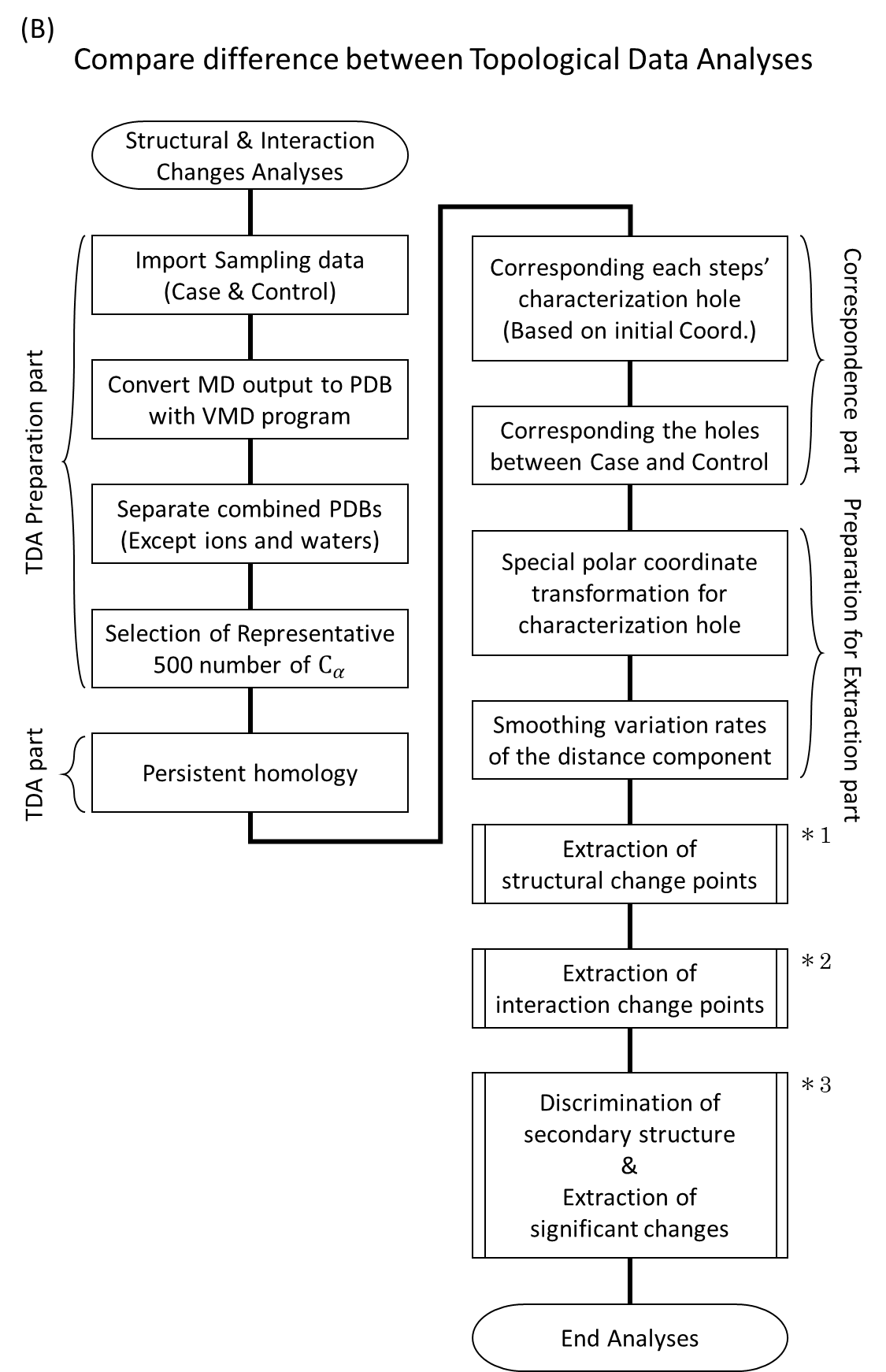


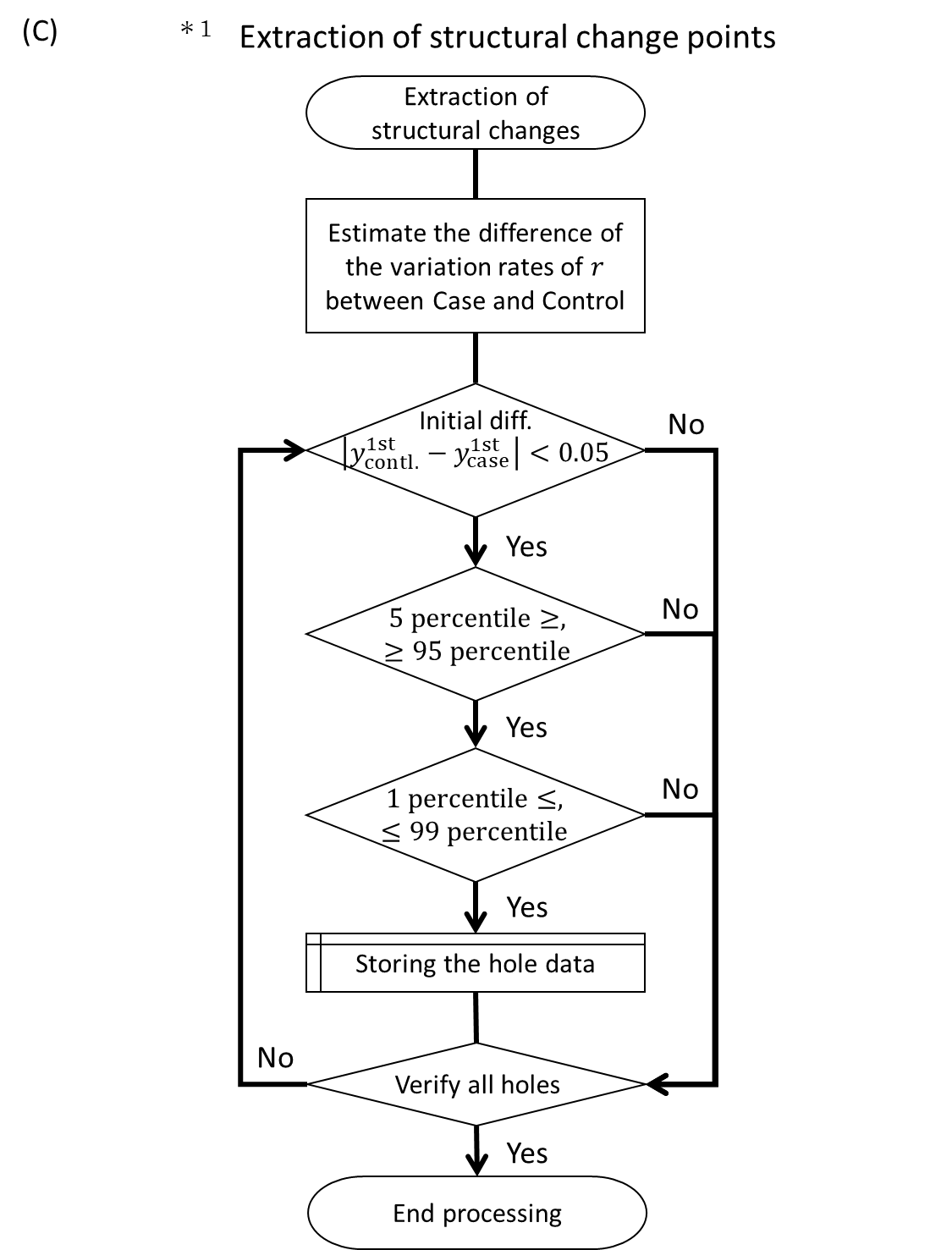


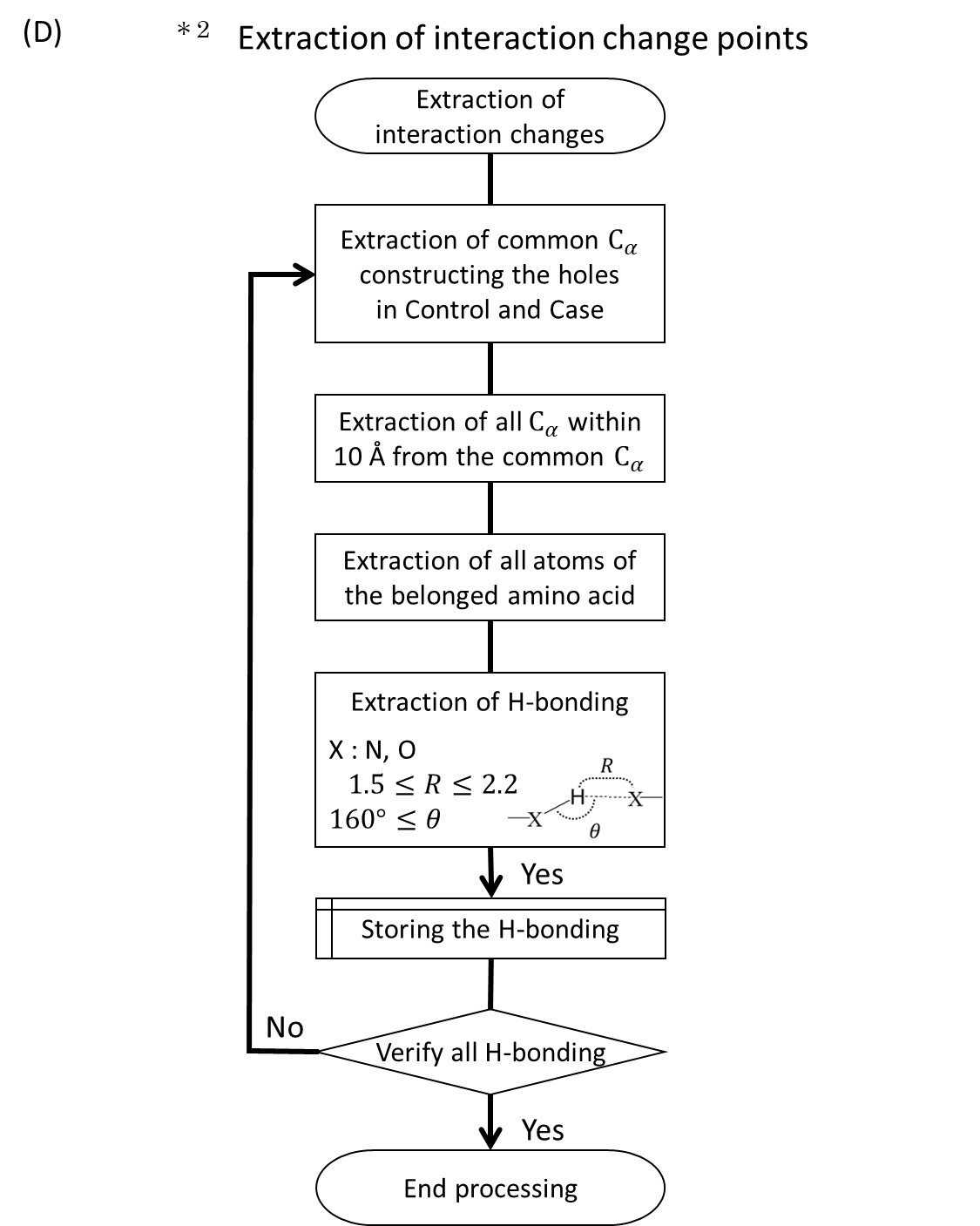


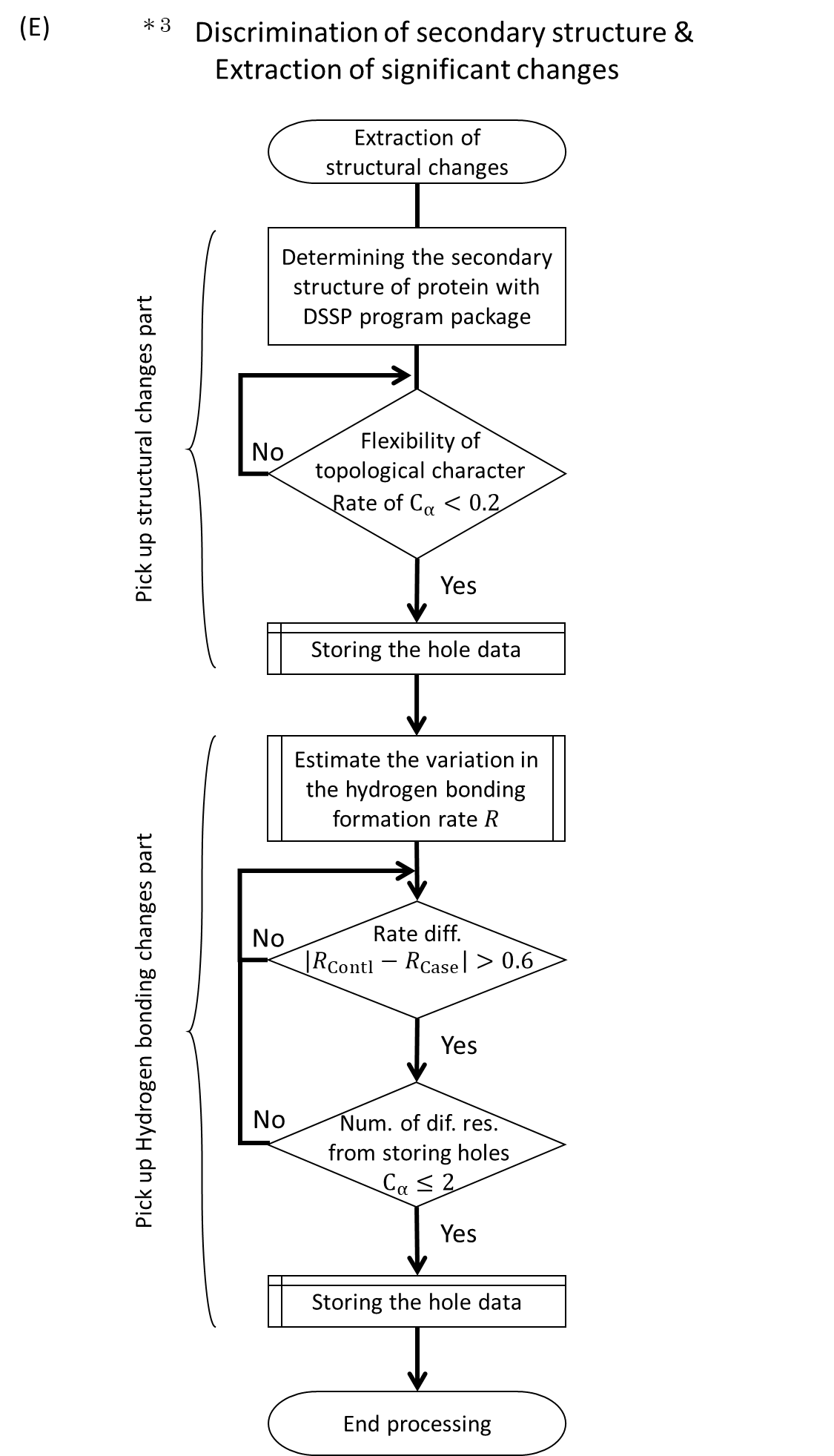


**Figure S8:** Flowchart of DAIS (Dynamical Analysis of Interaction and Structural changes). (A) Thermal structure sampling sites using molecular dynamics. (B) Extraction of structural changes and interaction sites using topological data analysis. Configuration parameters for determining (C) structural change and (D) hydrogen bonding changes. (E) Determination of protein secondary structure using the DSSP method and extraction of significant structural and hydrogen bond changes.

**Supplemental Tables**

**Supplemental Table 1:** The number of conformational change counts for each amino acid observed using the persistent homology method.

|  |  | BA1 | | | |  | BA2 | | | |  | BA2.75 | | | |  | BA5 | | | |
| --- | --- | --- | --- | --- | --- | --- | --- | --- | --- | --- | --- | --- | --- | --- | --- | --- | --- | --- | --- | --- |
|  |  | Pattern 1 | Pattern 2 | Pattern 3 | Pattern 4 |  | Pattern 1 | Pattern 2 | Pattern 3 | Pattern 4 |  | Pattern 1 | Pattern 2 | Pattern 3 | Pattern 4 |  | Pattern 1 | Pattern 2 | Pattern 3 | Pattern 4 |
| ILE | 332 | 0 | 0 | 0 | 0 |  | 0 | 0 | 0 | 1 |  | 0 | 0 | 0 | 0 |  | 0 | 0 | 0 | 0 |
| THR | 333 | 0 | 0 | 0 | 0 |  | 0 | 0 | 0 | 0 |  | 0 | 0 | 0 | 0 |  | 0 | 0 | 0 | 0 |
| ASN | 334 | 0 | 0 | 0 | 0 |  | 0 | 0 | 0 | 0 |  | 0 | 0 | 0 | 0 |  | 0 | 0 | 0 | 0 |
| LEU | 335 | 0 | 0 | 0 | 0 |  | 0 | 0 | 0 | 0 |  | 0 | 0 | 0 | 1 |  | 0 | 1 | 0 | 0 |
| CYS | 336 | 0 | 0 | 0 | 0 |  | 0 | 0 | 0 | 0 |  | 1 | 0 | 0 | 1 |  | 0 | 1 | 1 | 0 |
| PRO | 337 | 0 | 0 | 0 | 0 |  | 0 | 0 | 0 | 0 |  | 0 | 0 | 0 | 1 |  | 0 | 1 | 0 | 0 |
| PHE | 338 | 1 | 0 | 1 | 0 |  | 1 | 1 | 1 | 0 |  | 0 | 0 | 0 | 1 |  | 0 | 1 | 0 | 0 |
| GLY | 339 | 1 | 0 | 0 | 0 |  | 1 | 0 | 1 | 0 |  | 0 | 0 | 0 | 1 |  | 0 | 1 | 0 | 0 |
| GLU | 340 | 0 | 0 | 0 | 0 |  | 0 | 0 | 1 | 0 |  | 0 | 0 | 0 | 0 |  | 0 | 1 | 0 | 0 |
| VAL | 341 | 0 | 0 | 0 | 0 |  | 0 | 1 | 0 | 0 |  | 0 | 0 | 0 | 0 |  | 0 | 1 | 0 | 0 |
| PHE | 342 | 0 | 0 | 1 | 0 |  | 0 | 0 | 1 | 0 |  | 0 | 0 | 0 | 1 |  | 0 | 1 | 0 | 0 |
| ASN | 343 | 0 | 0 | 0 | 0 |  | 0 | 0 | 1 | 0 |  | 0 | 0 | 0 | 1 |  | 0 | 1 | 0 | 0 |
| ALA | 344 | 0 | 0 | 0 | 0 |  | 0 | 0 | 1 | 0 |  | 0 | 0 | 0 | 1 |  | 0 | 1 | 0 | 0 |
| THR | 345 | 0 | 0 | 0 | 0 |  | 0 | 0 | 0 | 0 |  | 0 | 0 | 0 | 1 |  | 0 | 1 | 0 | 0 |
| GLU | 346 | 0 | 0 | 0 | 0 |  | 0 | 0 | 0 | 0 |  | 0 | 0 | 0 | 1 |  | 0 | 1 | 0 | 0 |
| PHE | 347 | 0 | 0 | 0 | 0 |  | 0 | 0 | 0 | 0 |  | 0 | 0 | 0 | 1 |  | 0 | 1 | 0 | 0 |
| ALA | 348 | 0 | 0 | 0 | 0 |  | 0 | 0 | 0 | 0 |  | 0 | 0 | 0 | 1 |  | 0 | 1 | 0 | 1 |
| SER | 349 | 0 | 1 | 0 | 0 |  | 0 | 2 | 0 | 0 |  | 0 | 1 | 0 | 0 |  | 0 | 1 | 0 | 1 |
| VAL | 350 | 0 | 1 | 1 | 1 |  | 0 | 2 | 0 | 0 |  | 0 | 1 | 0 | 0 |  | 0 | 1 | 0 | 1 |
| TYR | 351 | 0 | 1 | 0 | 1 |  | 0 | 2 | 0 | 0 |  | 0 | 2 | 0 | 0 |  | 1 | 1 | 0 | 1 |
| ALA | 352 | 0 | 0 | 0 | 0 |  | 0 | 0 | 0 | 0 |  | 0 | 0 | 0 | 0 |  | 0 | 0 | 0 | 0 |
| TRP | 353 | 0 | 0 | 0 | 0 |  | 0 | 0 | 1 | 0 |  | 0 | 0 | 0 | 0 |  | 0 | 1 | 0 | 1 |
| ASN | 354 | 0 | 0 | 0 | 0 |  | 0 | 0 | 1 | 0 |  | 0 | 0 | 0 | 0 |  | 0 | 1 | 0 | 1 |
| ARG | 355 | 0 | 0 | 0 | 0 |  | 0 | 0 | 1 | 0 |  | 0 | 0 | 0 | 1 |  | 0 | 1 | 3 | 1 |
| LYS | 356 | 0 | 0 | 0 | 0 |  | 0 | 0 | 0 | 0 |  | 0 | 0 | 0 | 1 |  | 0 | 1 | 3 | 0 |
| ARG | 357 | 0 | 0 | 0 | 0 |  | 0 | 0 | 0 | 0 |  | 1 | 0 | 0 | 1 |  | 2 | 1 | 3 | 0 |
| ILE | 358 | 0 | 0 | 0 | 0 |  | 0 | 1 | 0 | 0 |  | 2 | 0 | 0 | 1 |  | 2 | 1 | 0 | 0 |
| SER | 359 | 0 | 0 | 0 | 0 |  | 0 | 0 | 0 | 1 |  | 2 | 0 | 0 | 1 |  | 1 | 2 | 0 | 0 |
| ASN | 360 | 0 | 0 | 0 | 0 |  | 0 | 1 | 1 | 0 |  | 1 | 0 | 0 | 1 |  | 1 | 2 | 0 | 0 |
| CYS | 361 | 0 | 0 | 0 | 0 |  | 0 | 1 | 1 | 1 |  | 1 | 0 | 0 | 1 |  | 1 | 2 | 1 | 0 |
| VAL | 362 | 0 | 0 | 0 | 0 |  | 0 | 1 | 1 | 0 |  | 0 | 0 | 0 | 0 |  | 1 | 2 | 1 | 0 |
| ALA | 363 | 0 | 0 | 0 | 0 |  | 0 | 1 | 0 | 0 |  | 0 | 1 | 0 | 0 |  | 1 | 2 | 1 | 0 |
| ASP | 364 | 0 | 0 | 0 | 0 |  | 0 | 0 | 0 | 0 |  | 0 | 1 | 0 | 0 |  | 1 | 1 | 0 | 0 |
| TYR | 365 | 0 | 0 | 0 | 0 |  | 0 | 1 | 0 | 0 |  | 0 | 1 | 0 | 0 |  | 1 | 1 | 1 | 0 |
| SER | 366 | 0 | 0 | 0 | 0 |  | 0 | 0 | 0 | 0 |  | 0 | 1 | 0 | 0 |  | 0 | 1 | 0 | 0 |
| VAL | 367 | 1 | 0 | 0 | 0 |  | 1 | 0 | 0 | 0 |  | 0 | 0 | 0 | 0 |  | 0 | 0 | 0 | 0 |
| LEU | 368 | 1 | 0 | 1 | 0 |  | 1 | 1 | 1 | 0 |  | 0 | 0 | 0 | 0 |  | 0 | 0 | 0 | 0 |
| TYR | 369 | 0 | 0 | 0 | 0 |  | 0 | 0 | 0 | 0 |  | 1 | 0 | 0 | 0 |  | 1 | 0 | 1 | 0 |
| ASN | 370 | 0 | 0 | 0 | 0 |  | 0 | 0 | 0 | 0 |  | 0 | 0 | 0 | 0 |  | 0 | 0 | 0 | 0 |
| SER | 371 | 1 | 0 | 1 | 0 |  | 1 | 0 | 1 | 0 |  | 1 | 0 | 0 | 0 |  | 1 | 0 | 0 | 0 |
| ALA | 372 | 0 | 0 | 0 | 0 |  | 0 | 0 | 0 | 0 |  | 1 | 0 | 0 | 0 |  | 1 | 0 | 0 | 0 |
| SER | 373 | 0 | 0 | 0 | 0 |  | 0 | 0 | 0 | 0 |  | 0 | 0 | 0 | 0 |  | 0 | 0 | 0 | 0 |
| PHE | 374 | 0 | 0 | 0 | 0 |  | 0 | 0 | 0 | 0 |  | 0 | 0 | 0 | 0 |  | 1 | 0 | 0 | 0 |
| SER | 375 | 0 | 0 | 0 | 0 |  | 0 | 0 | 0 | 0 |  | 0 | 0 | 0 | 0 |  | 0 | 0 | 0 | 0 |
| THR | 376 | 0 | 0 | 0 | 0 |  | 0 | 0 | 0 | 0 |  | 0 | 0 | 0 | 0 |  | 0 | 0 | 0 | 0 |
| PHE | 377 | 0 | 0 | 0 | 0 |  | 0 | 0 | 0 | 0 |  | 1 | 0 | 0 | 0 |  | 1 | 0 | 0 | 1 |
| LYS | 378 | 0 | 0 | 0 | 0 |  | 0 | 0 | 0 | 0 |  | 0 | 0 | 0 | 0 |  | 0 | 0 | 0 | 1 |
| CYS | 379 | 0 | 0 | 0 | 0 |  | 0 | 0 | 1 | 0 |  | 0 | 0 | 1 | 0 |  | 0 | 0 | 0 | 1 |
| TYR | 380 | 0 | 0 | 0 | 0 |  | 0 | 0 | 1 | 0 |  | 0 | 0 | 1 | 0 |  | 0 | 0 | 0 | 0 |
| GLY | 381 | 0 | 0 | 0 | 0 |  | 0 | 0 | 0 | 0 |  | 0 | 0 | 1 | 0 |  | 0 | 0 | 0 | 0 |
| VAL | 382 | 0 | 0 | 0 | 0 |  | 0 | 0 | 1 | 0 |  | 0 | 0 | 0 | 0 |  | 0 | 0 | 0 | 0 |
| SER | 383 | 0 | 0 | 0 | 0 |  | 0 | 0 | 1 | 0 |  | 0 | 0 | 0 | 0 |  | 0 | 0 | 0 | 0 |
| PRO | 384 | 0 | 0 | 0 | 0 |  | 0 | 0 | 0 | 0 |  | 0 | 0 | 0 | 0 |  | 0 | 0 | 1 | 0 |
| THR | 385 | 0 | 0 | 0 | 0 |  | 0 | 0 | 0 | 0 |  | 0 | 0 | 0 | 0 |  | 0 | 0 | 1 | 0 |
| LYS | 386 | 0 | 0 | 0 | 0 |  | 0 | 0 | 0 | 0 |  | 0 | 0 | 0 | 0 |  | 0 | 1 | 0 | 0 |
| LEU | 387 | 0 | 0 | 0 | 0 |  | 0 | 0 | 0 | 0 |  | 0 | 0 | 0 | 0 |  | 0 | 1 | 1 | 0 |
| ASN | 388 | 0 | 0 | 0 | 0 |  | 0 | 0 | 0 | 0 |  | 0 | 0 | 0 | 0 |  | 0 | 0 | 0 | 0 |
| ASP | 389 | 0 | 0 | 0 | 0 |  | 0 | 0 | 0 | 0 |  | 0 | 0 | 0 | 0 |  | 0 | 0 | 0 | 0 |
| LEU | 390 | 0 | 0 | 0 | 0 |  | 0 | 0 | 0 | 0 |  | 0 | 0 | 0 | 0 |  | 0 | 0 | 0 | 0 |
| CYS | 391 | 0 | 0 | 0 | 0 |  | 0 | 0 | 0 | 0 |  | 1 | 0 | 0 | 0 |  | 1 | 0 | 0 | 0 |
| PHE | 392 | 0 | 0 | 0 | 0 |  | 0 | 0 | 0 | 0 |  | 0 | 0 | 0 | 0 |  | 0 | 0 | 0 | 0 |
| THR | 393 | 0 | 0 | 0 | 0 |  | 0 | 0 | 0 | 0 |  | 2 | 0 | 0 | 0 |  | 1 | 0 | 0 | 0 |
| ASN | 394 | 0 | 0 | 0 | 0 |  | 0 | 0 | 0 | 0 |  | 2 | 0 | 0 | 0 |  | 1 | 1 | 0 | 0 |
| VAL | 395 | 0 | 0 | 0 | 1 |  | 0 | 0 | 0 | 0 |  | 2 | 0 | 0 | 0 |  | 2 | 0 | 0 | 0 |
| TYR | 396 | 0 | 0 | 0 | 1 |  | 0 | 0 | 0 | 0 |  | 1 | 0 | 0 | 0 |  | 2 | 0 | 3 | 0 |
| ALA | 397 | 0 | 0 | 0 | 1 |  | 0 | 1 | 0 | 0 |  | 0 | 0 | 0 | 0 |  | 0 | 0 | 3 | 0 |
| ASP | 398 | 0 | 0 | 0 | 1 |  | 0 | 0 | 1 | 1 |  | 0 | 0 | 0 | 0 |  | 0 | 0 | 3 | 1 |
| SER | 399 | 0 | 0 | 0 | 1 |  | 0 | 0 | 0 | 1 |  | 0 | 0 | 0 | 0 |  | 0 | 0 | 0 | 1 |
| PHE | 400 | 0 | 0 | 0 | 1 |  | 0 | 0 | 0 | 1 |  | 0 | 0 | 0 | 0 |  | 0 | 0 | 0 | 1 |
| VAL | 401 | 0 | 0 | 0 | 1 |  | 0 | 0 | 0 | 1 |  | 0 | 0 | 0 | 0 |  | 0 | 0 | 0 | 0 |
| ILE | 402 | 0 | 0 | 0 | 1 |  | 0 | 0 | 0 | 1 |  | 0 | 0 | 0 | 0 |  | 0 | 0 | 0 | 1 |
| ARG | 403 | 1 | 0 | 0 | 1 |  | 1 | 1 | 0 | 1 |  | 0 | 0 | 0 | 0 |  | 0 | 0 | 0 | 0 |
| GLY | 404 | 1 | 0 | 0 | 1 |  | 1 | 1 | 0 | 1 |  | 0 | 0 | 0 | 0 |  | 0 | 0 | 0 | 0 |
| ASP | 405 | 0 | 0 | 0 | 0 |  | 0 | 1 | 0 | 1 |  | 0 | 0 | 0 | 0 |  | 0 | 0 | 0 | 0 |
| GLU | 406 | 0 | 0 | 0 | 0 |  | 0 | 0 | 0 | 1 |  | 0 | 0 | 0 | 0 |  | 0 | 0 | 0 | 0 |
| VAL | 407 | 0 | 0 | 0 | 0 |  | 0 | 0 | 0 | 0 |  | 0 | 0 | 0 | 0 |  | 0 | 0 | 0 | 1 |
| ARG | 408 | 0 | 0 | 0 | 0 |  | 0 | 0 | 0 | 0 |  | 0 | 0 | 0 | 0 |  | 0 | 0 | 0 | 0 |
| GLN | 409 | 0 | 0 | 0 | 0 |  | 0 | 0 | 0 | 1 |  | 0 | 1 | 0 | 0 |  | 0 | 0 | 0 | 0 |
| ILE | 410 | 0 | 0 | 0 | 0 |  | 0 | 2 | 0 | 2 |  | 0 | 0 | 0 | 0 |  | 0 | 0 | 0 | 1 |
| ALA | 411 | 0 | 0 | 0 | 0 |  | 0 | 1 | 0 | 1 |  | 0 | 0 | 0 | 0 |  | 0 | 0 | 0 | 0 |
| PRO | 412 | 0 | 0 | 0 | 0 |  | 0 | 0 | 0 | 1 |  | 0 | 1 | 0 | 0 |  | 0 | 0 | 0 | 0 |
| GLY | 413 | 0 | 0 | 0 | 0 |  | 0 | 0 | 0 | 1 |  | 1 | 0 | 0 | 0 |  | 0 | 0 | 1 | 0 |
| GLN | 414 | 0 | 0 | 0 | 0 |  | 0 | 0 | 0 | 1 |  | 1 | 1 | 0 | 0 |  | 0 | 0 | 1 | 0 |
| THR | 415 | 0 | 0 | 0 | 0 |  | 0 | 0 | 0 | 1 |  | 1 | 1 | 0 | 0 |  | 0 | 0 | 1 | 0 |
| GLY | 416 | 0 | 0 | 0 | 0 |  | 0 | 1 | 0 | 1 |  | 1 | 0 | 0 | 0 |  | 0 | 0 | 1 | 0 |
| LYS | 417 | 0 | 0 | 0 | 0 |  | 0 | 1 | 0 | 1 |  | 1 | 0 | 0 | 0 |  | 0 | 0 | 1 | 0 |
| ILE | 418 | 0 | 0 | 0 | 0 |  | 0 | 0 | 0 | 1 |  | 1 | 0 | 0 | 0 |  | 0 | 0 | 1 | 0 |
| ALA | 419 | 0 | 0 | 1 | 0 |  | 0 | 1 | 0 | 2 |  | 1 | 1 | 0 | 0 |  | 0 | 0 | 1 | 0 |
| ASP | 420 | 0 | 0 | 1 | 0 |  | 0 | 0 | 0 | 1 |  | 1 | 0 | 0 | 0 |  | 1 | 0 | 1 | 0 |
| TYR | 421 | 0 | 0 | 3 | 0 |  | 0 | 1 | 1 | 1 |  | 1 | 1 | 0 | 0 |  | 1 | 0 | 1 | 0 |
| ASN | 422 | 0 | 0 | 4 | 1 |  | 0 | 1 | 1 | 2 |  | 1 | 1 | 0 | 0 |  | 1 | 0 | 1 | 0 |
| TYR | 423 | 0 | 1 | 2 | 1 |  | 0 | 1 | 0 | 3 |  | 1 | 0 | 0 | 0 |  | 0 | 0 | 1 | 0 |
| LYS | 424 | 0 | 1 | 0 | 0 |  | 0 | 2 | 0 | 1 |  | 1 | 0 | 0 | 0 |  | 0 | 0 | 1 | 0 |
| LEU | 425 | 0 | 0 | 0 | 0 |  | 0 | 1 | 0 | 0 |  | 1 | 1 | 0 | 0 |  | 0 | 0 | 1 | 0 |
| PRO | 426 | 0 | 0 | 0 | 0 |  | 0 | 0 | 0 | 0 |  | 1 | 1 | 0 | 0 |  | 0 | 0 | 1 | 0 |
| ASP | 427 | 0 | 0 | 0 | 0 |  | 0 | 0 | 0 | 0 |  | 1 | 1 | 0 | 0 |  | 0 | 0 | 1 | 0 |
| ASP | 428 | 0 | 0 | 0 | 0 |  | 0 | 0 | 0 | 0 |  | 0 | 0 | 0 | 0 |  | 0 | 0 | 0 | 0 |
| PHE | 429 | 0 | 0 | 0 | 0 |  | 0 | 0 | 0 | 0 |  | 0 | 0 | 0 | 0 |  | 0 | 0 | 0 | 0 |
| THR | 430 | 0 | 0 | 0 | 0 |  | 0 | 0 | 0 | 0 |  | 0 | 0 | 1 | 0 |  | 0 | 0 | 0 | 0 |
| GLY | 431 | 1 | 0 | 0 | 0 |  | 1 | 0 | 0 | 0 |  | 0 | 0 | 1 | 0 |  | 0 | 0 | 0 | 0 |
| CYS | 432 | 1 | 0 | 0 | 0 |  | 1 | 0 | 0 | 0 |  | 0 | 0 | 1 | 0 |  | 0 | 0 | 0 | 1 |
| VAL | 433 | 1 | 0 | 0 | 0 |  | 1 | 0 | 0 | 0 |  | 0 | 0 | 0 | 0 |  | 0 | 0 | 0 | 2 |
| ILE | 434 | 1 | 0 | 0 | 0 |  | 1 | 1 | 0 | 0 |  | 0 | 1 | 0 | 0 |  | 0 | 1 | 0 | 1 |
| ALA | 435 | 1 | 0 | 0 | 0 |  | 1 | 0 | 0 | 0 |  | 0 | 1 | 0 | 0 |  | 0 | 1 | 1 | 0 |
| TRP | 436 | 1 | 0 | 0 | 0 |  | 1 | 0 | 0 | 0 |  | 0 | 0 | 0 | 0 |  | 0 | 0 | 1 | 0 |
| ASN | 437 | 1 | 0 | 0 | 0 |  | 1 | 0 | 0 | 0 |  | 0 | 0 | 0 | 0 |  | 0 | 0 | 1 | 0 |
| SER | 438 | 1 | 0 | 0 | 0 |  | 1 | 0 | 0 | 0 |  | 0 | 0 | 0 | 0 |  | 0 | 0 | 1 | 0 |
| ASN | 439 | 1 | 0 | 0 | 0 |  | 1 | 0 | 0 | 0 |  | 0 | 0 | 0 | 0 |  | 0 | 0 | 1 | 1 |
| ASN | 440 | 1 | 0 | 0 | 1 |  | 1 | 0 | 0 | 0 |  | 0 | 0 | 0 | 0 |  | 0 | 0 | 1 | 2 |
| LEU | 441 | 1 | 0 | 0 | 1 |  | 1 | 0 | 0 | 0 |  | 0 | 0 | 0 | 0 |  | 0 | 0 | 1 | 1 |
| ASP | 442 | 1 | 0 | 0 | 0 |  | 1 | 0 | 0 | 0 |  | 0 | 0 | 0 | 0 |  | 0 | 0 | 1 | 1 |
| SER | 443 | 1 | 0 | 0 | 0 |  | 1 | 0 | 0 | 0 |  | 0 | 0 | 0 | 1 |  | 0 | 0 | 1 | 1 |
| LYS | 444 | 1 | 0 | 0 | 0 |  | 1 | 0 | 1 | 0 |  | 0 | 0 | 1 | 1 |  | 0 | 0 | 1 | 2 |
| VAL | 445 | 1 | 0 | 0 | 0 |  | 1 | 0 | 1 | 0 |  | 0 | 0 | 2 | 1 |  | 0 | 0 | 0 | 0 |
| GLY | 446 | 1 | 0 | 0 | 0 |  | 1 | 0 | 2 | 0 |  | 0 | 1 | 2 | 1 |  | 0 | 0 | 0 | 0 |
| GLY | 447 | 1 | 0 | 0 | 0 |  | 1 | 0 | 2 | 0 |  | 0 | 1 | 3 | 1 |  | 0 | 0 | 0 | 0 |
| ASN | 448 | 1 | 0 | 0 | 0 |  | 1 | 0 | 0 | 0 |  | 0 | 1 | 2 | 0 |  | 0 | 0 | 0 | 0 |
| TYR | 449 | 1 | 0 | 0 | 0 |  | 1 | 0 | 0 | 0 |  | 0 | 0 | 1 | 1 |  | 0 | 0 | 1 | 0 |
| ASN | 450 | 1 | 0 | 0 | 0 |  | 1 | 0 | 0 | 0 |  | 0 | 0 | 1 | 1 |  | 0 | 0 | 1 | 0 |
| TYR | 451 | 1 | 1 | 0 | 0 |  | 1 | 2 | 0 | 1 |  | 0 | 0 | 1 | 1 |  | 0 | 0 | 1 | 0 |
| LEU | 452 | 1 | 1 | 0 | 0 |  | 1 | 2 | 0 | 1 |  | 0 | 0 | 1 | 1 |  | 0 | 0 | 1 | 0 |
| TYR | 453 | 1 | 1 | 2 | 0 |  | 1 | 2 | 1 | 1 |  | 0 | 0 | 0 | 0 |  | 1 | 0 | 0 | 0 |
| ARG | 454 | 1 | 0 | 0 | 1 |  | 1 | 0 | 0 | 1 |  | 0 | 2 | 1 | 0 |  | 1 | 0 | 1 | 0 |
| LEU | 455 | 1 | 0 | 1 | 1 |  | 1 | 0 | 1 | 1 |  | 0 | 2 | 0 | 0 |  | 1 | 0 | 0 | 0 |
| PHE | 456 | 1 | 0 | 0 | 0 |  | 1 | 0 | 0 | 1 |  | 0 | 1 | 1 | 0 |  | 1 | 0 | 1 | 0 |
| ARG | 457 | 1 | 0 | 0 | 0 |  | 1 | 0 | 0 | 1 |  | 0 | 0 | 0 | 0 |  | 1 | 0 | 0 | 0 |
| LYS | 458 | 1 | 0 | 0 | 0 |  | 1 | 0 | 0 | 1 |  | 0 | 0 | 0 | 0 |  | 1 | 0 | 0 | 0 |
| SER | 459 | 1 | 0 | 0 | 0 |  | 1 | 1 | 0 | 1 |  | 0 | 0 | 0 | 0 |  | 2 | 0 | 0 | 0 |
| ASN | 460 | 1 | 0 | 0 | 0 |  | 1 | 1 | 0 | 1 |  | 0 | 0 | 0 | 0 |  | 1 | 0 | 0 | 0 |
| LEU | 461 | 1 | 0 | 1 | 0 |  | 1 | 1 | 0 | 1 |  | 0 | 0 | 0 | 0 |  | 0 | 0 | 0 | 0 |
| LYS | 462 | 1 | 0 | 0 | 0 |  | 1 | 0 | 0 | 1 |  | 0 | 0 | 0 | 0 |  | 0 | 0 | 0 | 0 |
| PRO | 463 | 1 | 1 | 1 | 0 |  | 1 | 1 | 0 | 1 |  | 0 | 0 | 0 | 0 |  | 0 | 0 | 0 | 0 |
| PHE | 464 | 1 | 1 | 0 | 0 |  | 1 | 0 | 0 | 2 |  | 0 | 0 | 0 | 0 |  | 0 | 0 | 0 | 0 |
| GLU | 465 | 1 | 0 | 1 | 1 |  | 1 | 0 | 0 | 1 |  | 0 | 0 | 0 | 0 |  | 0 | 0 | 0 | 0 |
| ARG | 466 | 1 | 0 | 0 | 0 |  | 1 | 0 | 0 | 0 |  | 0 | 0 | 0 | 0 |  | 0 | 0 | 0 | 0 |
| ASP | 467 | 1 | 0 | 0 | 0 |  | 1 | 0 | 0 | 0 |  | 0 | 0 | 0 | 0 |  | 0 | 0 | 0 | 0 |
| ILE | 468 | 1 | 0 | 0 | 0 |  | 1 | 0 | 0 | 0 |  | 0 | 0 | 0 | 0 |  | 0 | 0 | 0 | 0 |
| SER | 469 | 1 | 0 | 0 | 0 |  | 1 | 0 | 0 | 0 |  | 0 | 0 | 0 | 0 |  | 0 | 0 | 0 | 0 |
| THR | 470 | 2 | 0 | 0 | 0 |  | 2 | 0 | 0 | 0 |  | 0 | 0 | 0 | 0 |  | 0 | 0 | 0 | 0 |
| GLU | 471 | 2 | 0 | 0 | 0 |  | 2 | 0 | 0 | 0 |  | 0 | 0 | 0 | 0 |  | 0 | 0 | 0 | 0 |
| ILE | 472 | 2 | 0 | 0 | 0 |  | 2 | 0 | 0 | 0 |  | 0 | 1 | 0 | 0 |  | 1 | 0 | 1 | 0 |
| TYR | 473 | 1 | 0 | 1 | 0 |  | 1 | 0 | 0 | 0 |  | 1 | 1 | 1 | 0 |  | 0 | 0 | 1 | 0 |
| GLN | 474 | 1 | 0 | 1 | 0 |  | 1 | 0 | 0 | 0 |  | 1 | 0 | 1 | 0 |  | 0 | 0 | 1 | 0 |
| ALA | 475 | 1 | 0 | 0 | 0 |  | 1 | 0 | 0 | 0 |  | 2 | 0 | 0 | 0 |  | 0 | 0 | 0 | 0 |
| GLY | 476 | 1 | 0 | 0 | 0 |  | 1 | 0 | 0 | 0 |  | 1 | 0 | 0 | 0 |  | 0 | 0 | 0 | 0 |
| SER | 477 | 1 | 0 | 0 | 0 |  | 1 | 0 | 0 | 0 |  | 1 | 0 | 0 | 0 |  | 0 | 0 | 1 | 0 |
| THR | 478 | 1 | 0 | 0 | 0 |  | 1 | 0 | 0 | 0 |  | 1 | 0 | 0 | 0 |  | 0 | 0 | 0 | 0 |
| PRO | 479 | 1 | 0 | 0 | 0 |  | 1 | 0 | 0 | 0 |  | 1 | 0 | 0 | 0 |  | 0 | 0 | 0 | 1 |
| CYS | 480 | 1 | 0 | 0 | 0 |  | 1 | 0 | 1 | 0 |  | 2 | 0 | 0 | 0 |  | 0 | 0 | 0 | 1 |
| ASN | 481 | 1 | 0 | 0 | 0 |  | 1 | 0 | 1 | 0 |  | 2 | 0 | 0 | 0 |  | 0 | 0 | 0 | 1 |
| GLY | 482 | 1 | 0 | 0 | 0 |  | 1 | 0 | 1 | 0 |  | 2 | 0 | 0 | 0 |  | 1 | 0 | 0 | 1 |
| VAL | 483 | 1 | 0 | 0 | 0 |  | 1 | 0 | 1 | 0 |  | 2 | 1 | 0 | 0 |  | 1 | 0 | 1 | 0 |
| GLU | 484 | 1 | 0 | 0 | 0 |  | 1 | 0 | 1 | 0 |  | 1 | 1 | 0 | 0 |  | 1 | 1 | 2 | 0 |
| GLY | 485 | 1 | 0 | 0 | 0 |  | 1 | 0 | 0 | 0 |  | 1 | 0 | 0 | 0 |  | 0 | 1 | 1 | 0 |
| PHE | 486 | 1 | 0 | 0 | 0 |  | 1 | 0 | 0 | 0 |  | 1 | 1 | 0 | 0 |  | 0 | 1 | 1 | 0 |
| ASN | 487 | 1 | 0 | 0 | 0 |  | 1 | 0 | 0 | 0 |  | 1 | 1 | 0 | 0 |  | 0 | 0 | 1 | 0 |
| CYS | 488 | 1 | 0 | 1 | 0 |  | 1 | 0 | 0 | 0 |  | 2 | 2 | 0 | 0 |  | 0 | 0 | 1 | 0 |
| TYR | 489 | 1 | 0 | 1 | 1 |  | 1 | 0 | 0 | 0 |  | 0 | 0 | 0 | 0 |  | 0 | 0 | 0 | 1 |
| PHE | 490 | 2 | 0 | 0 | 1 |  | 2 | 0 | 0 | 0 |  | 0 | 1 | 0 | 0 |  | 0 | 0 | 0 | 0 |
| PRO | 491 | 2 | 0 | 0 | 0 |  | 2 | 0 | 0 | 0 |  | 0 | 1 | 1 | 0 |  | 0 | 0 | 1 | 0 |
| LEU | 492 | 1 | 0 | 0 | 0 |  | 1 | 0 | 0 | 0 |  | 0 | 1 | 0 | 0 |  | 0 | 0 | 0 | 0 |
| GLN | 493 | 1 | 0 | 0 | 0 |  | 1 | 0 | 0 | 0 |  | 0 | 0 | 0 | 0 |  | 0 | 0 | 0 | 0 |
| SER | 494 | 1 | 0 | 0 | 0 |  | 1 | 0 | 0 | 0 |  | 0 | 0 | 1 | 1 |  | 0 | 0 | 1 | 0 |
| TYR | 495 | 1 | 0 | 0 | 0 |  | 1 | 0 | 0 | 0 |  | 0 | 0 | 1 | 0 |  | 0 | 0 | 1 | 0 |
| GLY | 496 | 1 | 0 | 0 | 0 |  | 1 | 0 | 1 | 0 |  | 0 | 0 | 1 | 0 |  | 0 | 0 | 1 | 0 |
| PHE | 497 | 1 | 0 | 0 | 0 |  | 1 | 0 | 1 | 0 |  | 0 | 1 | 1 | 0 |  | 0 | 0 | 0 | 0 |
| GLN | 498 | 1 | 0 | 0 | 0 |  | 1 | 0 | 0 | 0 |  | 0 | 1 | 2 | 0 |  | 0 | 0 | 0 | 0 |
| PRO | 499 | 1 | 0 | 0 | 0 |  | 1 | 0 | 0 | 0 |  | 0 | 0 | 1 | 0 |  | 0 | 0 | 1 | 0 |
| THR | 500 | 1 | 0 | 0 | 0 |  | 1 | 0 | 0 | 0 |  | 0 | 0 | 0 | 0 |  | 0 | 0 | 1 | 0 |
| ASN | 501 | 1 | 0 | 0 | 0 |  | 1 | 0 | 0 | 0 |  | 0 | 0 | 0 | 0 |  | 0 | 0 | 1 | 0 |
| GLY | 502 | 1 | 0 | 0 | 0 |  | 1 | 0 | 0 | 0 |  | 0 | 0 | 0 | 0 |  | 0 | 0 | 1 | 0 |
| VAL | 503 | 1 | 0 | 0 | 0 |  | 1 | 0 | 0 | 0 |  | 0 | 0 | 0 | 0 |  | 0 | 0 | 1 | 0 |
| GLY | 504 | 1 | 0 | 0 | 1 |  | 1 | 0 | 0 | 0 |  | 0 | 0 | 0 | 0 |  | 0 | 0 | 1 | 0 |
| TYR | 505 | 2 | 0 | 0 | 1 |  | 2 | 1 | 0 | 0 |  | 0 | 0 | 0 | 0 |  | 0 | 0 | 1 | 0 |
| GLN | 506 | 2 | 0 | 0 | 1 |  | 2 | 0 | 0 | 0 |  | 0 | 0 | 0 | 0 |  | 0 | 0 | 1 | 0 |
| PRO | 507 | 2 | 0 | 0 | 1 |  | 2 | 0 | 0 | 0 |  | 0 | 0 | 0 | 0 |  | 0 | 0 | 1 | 0 |
| TYR | 508 | 2 | 0 | 0 | 1 |  | 2 | 0 | 0 | 0 |  | 0 | 0 | 0 | 0 |  | 0 | 0 | 1 | 0 |
| ARG | 509 | 1 | 0 | 0 | 1 |  | 1 | 0 | 0 | 0 |  | 0 | 0 | 0 | 0 |  | 0 | 0 | 1 | 0 |
| VAL | 510 | 1 | 0 | 0 | 1 |  | 1 | 0 | 0 | 0 |  | 0 | 1 | 0 | 0 |  | 0 | 1 | 1 | 1 |
| VAL | 511 | 1 | 0 | 0 | 1 |  | 1 | 1 | 0 | 0 |  | 0 | 1 | 0 | 0 |  | 0 | 1 | 0 | 0 |
| VAL | 512 | 1 | 0 | 0 | 1 |  | 1 | 0 | 0 | 0 |  | 0 | 0 | 0 | 0 |  | 0 | 0 | 0 | 0 |
| LEU | 513 | 1 | 0 | 0 | 1 |  | 1 | 1 | 0 | 0 |  | 0 | 0 | 0 | 0 |  | 0 | 0 | 0 | 0 |
| SER | 514 | 1 | 0 | 0 | 1 |  | 1 | 0 | 0 | 0 |  | 0 | 0 | 0 | 0 |  | 0 | 0 | 0 | 0 |
| PHE | 515 | 0 | 0 | 0 | 1 |  | 0 | 0 | 0 | 0 |  | 1 | 0 | 0 | 0 |  | 0 | 0 | 0 | 0 |
| GLU | 516 | 0 | 0 | 0 | 0 |  | 0 | 0 | 0 | 0 |  | 1 | 0 | 0 | 0 |  | 0 | 0 | 0 | 0 |
| LEU | 517 | 0 | 0 | 0 | 0 |  | 0 | 0 | 0 | 0 |  | 1 | 0 | 0 | 0 |  | 0 | 0 | 0 | 0 |
| LEU | 518 | 0 | 0 | 0 | 0 |  | 0 | 0 | 0 | 0 |  | 0 | 0 | 0 | 0 |  | 0 | 0 | 0 | 0 |

**Supplemental Table 2:** The RBD-ACE2 binding structural class presented by Allison J. Greaney *et al.*

|  | PDB ID used in this study |
| --- | --- |
| Case 1 antibodies | 6XCM, 6XCN |
| Case 2 antibodies | 7K90, 7K8S, 7K8T, 7K8X, 7K8Y |
| Case 3 antibodies | 7K8Z, 7K8V |
